# Effective cell membrane tension protects red blood cells against malaria invasion

**DOI:** 10.1371/journal.pcbi.1011694

**Published:** 2023-12-04

**Authors:** Haleh Alimohamadi, Padmini Rangamani

**Affiliations:** Department of Mechanical and Aerospace Engineering, University of California San Diego, La Jolla, California, United States of America; Universitat des Saarlandes, GERMANY

## Abstract

A critical step in how malaria parasites invade red blood cells (RBCs) is the wrapping of the membrane around the egg-shaped merozoites. Recent experiments have revealed that RBCs can be protected from malaria invasion by high membrane tension. While cellular and biochemical aspects of parasite actomyosin motor forces during the malaria invasion have been well studied, the important role of the biophysical forces induced by the RBC membrane-cytoskeleton composite has not yet been fully understood. In this study, we use a theoretical model for lipid bilayer mechanics, cytoskeleton deformation, and membrane-merozoite interactions to systematically investigate the influence of effective RBC membrane tension, which includes contributions from the lipid bilayer tension, spontaneous tension, interfacial tension, and the resistance of cytoskeleton against shear deformation on the progression of membrane wrapping during the process of malaria invasion. Our model reveals that this effective membrane tension creates a wrapping energy barrier for a complete merozoite entry. We calculate the tension threshold required to impede the malaria invasion. We find that the tension threshold is a nonmonotonic function of spontaneous tension and undergoes a sharp transition from large to small values as the magnitude of interfacial tension increases. We also predict that the physical properties of the RBC cytoskeleton layer—particularly the resting length of the cytoskeleton—play key roles in specifying the degree of the membrane wrapping. We also found that the shear energy of cytoskeleton deformation diverges at the full wrapping state, suggesting the local disassembly of the cytoskeleton is required to complete the merozoite entry. Additionally, using our theoretical framework, we predict the landscape of myosin-mediated forces and the physical properties of the RBC membrane in regulating successful malaria invasion. Our findings on the crucial role of RBC membrane tension in inhibiting malaria invasion can have implications for developing novel antimalarial therapeutic or vaccine-based strategies.

## Author summary

RBC membrane tension plays an important role in regulating RBC shape and functionality. In particular, recent experimental studies have shown that elevated RBC membrane tension protects against severe malaria infection. In this study, we sought to identify how different contributions to the effective membrane tension can contribute to this mechanically driven protection against malaria invasion. Using a mathematical model, we derived a relationship between the effective tension of the RBC membrane—comprising a lipid bilayer and a cytoskeleton layer– and the degree of membrane wrapping during malaria invasion. Our model shows that the shear resistance of the RBC cytoskeleton plays an important role in inhibiting malaria invasion. Our findings can be generalized to the role of cell membrane mechanics in many wrapping phenomena providing insight into the crucial contributions of the host-cell membrane in protection against severe infections.

## Introduction

Malaria is one of the major infectious diseases and causes nearly half a million deaths per year worldwide [1]. Merozoites, which are protozoan parasites of the *Plasmodium* family, are small egg-shaped parasites with a diameter of 1–2 µm and are key to infecting red blood cells (RBCs) in the progression of malaria [2–4]. Merozoites invade healthy RBCs and asexually reproduce inside them; this is a critical step in the survival and reproduction of the parasites. In recent years, extensive studies have been focused on understanding the molecular and biophysical mechanisms underlying erythrocyte invasion as a route to develop novel antimalarial therapeutic or vaccine-based strategies [5–7].

**Fig 1:**
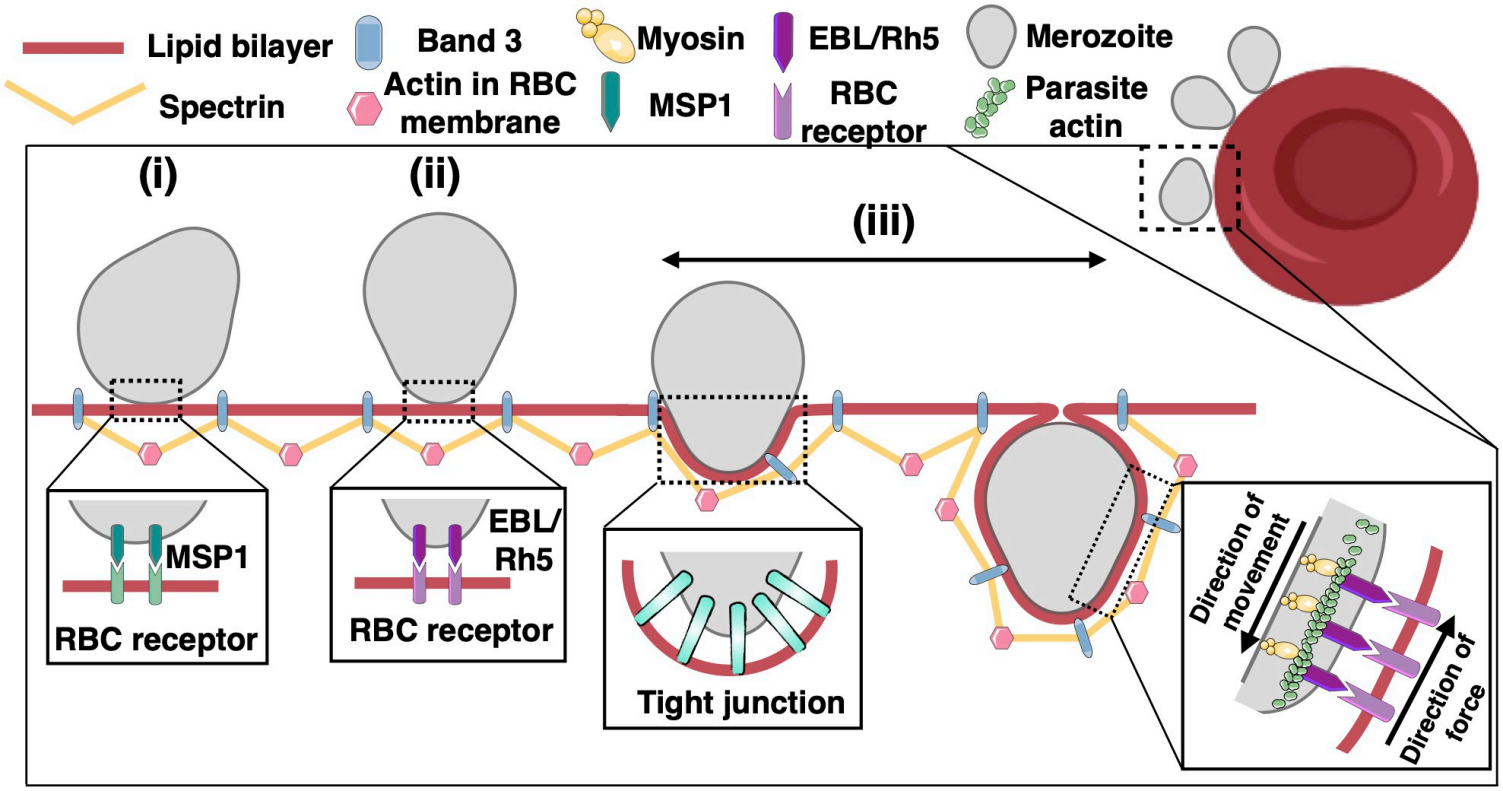
Schematic depiction of the molecular machinery of erythrocytic invasion by malaria parasites. Different stages of malaria invasion. The activity of the parasite actomyosin motors generates forces that push the merozoite into the RBC. In this study, we only focus on the erythrocyte membrane wrapping stage (iii).

At the cellular level, the invasion of erythrocytes by merozoites can be classified by the biophysical processes that include parasite binding to the RBC and subsequent membrane bending (Fig 1). The formation of the tight junction between the merozoite and the RBC involves low-affinity attachment of merozoite surface proteins 1 (MSP1) to RBCs, reorientation of the merozoite, and strengthening of the adhesion by binding of the erythrocyte-binding- like (EBL) or the reticulocyte binding antigen homolog (Rh) proteins and the erythrocyte membrane receptors (Fig 1) [8–10]. For a period of time, the penetration of the parasite into the RBC was assumed to be solely driven by the parasite actomyosin motors suggesting that the erythrocyte surface played a barrier role in the entire invasion (Fig 1) [11–14]. However, recent evidence has challenged this view [15–17]. Several studies have demonstrated the reorientation and the formation of host cell actin filaments dense structures at the point of entry during *Toxoplasma* and nonerythroid *Plasmodium* invasion [18–20]. Andenmatten et al. reported a degree of invasion even by knocking out the myosin and actin in *Toxoplasma*, proposing the existence of alternative invasion pathways in apicomplexan parasites [21]. Thus, all of these studies highlight the important role of the host cell during merozoite entry.

In this study, we specifically focus on the role of the RBC membrane tension on the invasion capability of the merozoite using a continuum mechanics approach. Our study is motivated by recent evidence that the tension of the RBC membrane has been implicated as a key determinant of merozoite invasion [22]. We first identify the known main contributors to the RBC membrane tension and RBC mechanical properties from the literature and summarize their role in the success or failure of the invasion below.

1. **Role of lipid bilayer inextensibility**: The lipid bilayer in an RBC is assumed to be resistant to stretch and, therefore, areally inextensibile. [23, 24]. For simplicity, here we exclude any mechanisms that could buffer membrane stretch such as membrane invaginations or exchange of lipids with the cytoplasm [25]. Mathematically, the tension of the bilayer is equivalent to the Lagrange multiplier used to maintain this inextensibility [26–28]. Several studies have shown that the rare Dantu variant of the glycophorin A/B receptors, which is associated with increased RBC tension, can protect against severe malaria [22, 29, 30]. Particularly, a recent experimental work by Kariuki et al. has shown the direct relationship between RBC tension and the efficiency of merozoite invasion [22]. Using the membrane flickering spectrometry technique, they demonstrated that Dantu RBCs with high tension deform less in contact with merozoites, and there is a tension threshold (< 3.8 ± 2 × 10^−7^ N/m) above which no invasion can take place [22]. Similar observations have been made about the increased susceptibility of young RBCs with lower tension to parasite *P. falciparum* invasion [31].
2. **Role of the RBC cytoskeleton:** The RBC cytoskeleton is a two dimensional lattice that is made of short F-actins interconnected by flexible spectrin molecules and provides support for the RBC membrane to maintain its curvature, tension, and physical properties [32–35]. The RBC cytoskeleton is a strong elastic network that restricts the deformation of the membrane and also contributes to the organization of the membrane proteins. Previous studies have shown that the bilayer-cytoskeleton interactions result in an effective membrane tension that is much larger (4–24 times greater) than that of vesicles without a cytoskeletal layer. [36, 37]. Thus, the mechanical properties of the RBC cytoskeleton and its interaction with the lipid bilayer play important roles in malaria invasion [13, 16]. For example, several studies have identified that in an ovalocytic erythrocyte, a more rigid cytoskeleton (3–4 times higher shear modulus compared to normal cells) significantly impairs the parasite invasion process [38–40]. *In vivo*, augmented RBCs with a cytosolic polyamine (e.g., spermine) demonstrated strong resistance against malaria invasion [41]. This is because adding polyamines increases the cohesion of the cytoskeleton and, ultimately, the mechanical rigidity of the whole RBC membrane [41]. Additionally, changes induced in the cytoskeleton structure and the viscoelastic properties of the RBC membrane due to phosphorylation of transmembrane and cytoskeletal erythrocyte proteins have been shown to facilitate malaria entry [13, 16, 42].
3. **Role of parasite-induced spontaneous tension:** Local protein-mediated adhesion of merozoites to the surface of RBCs can induce asymmetry in lipid orientation (including lipid tilt) and distribution such as the formation of lipid rafts [43–45]. This asymmetry in the lipid membrane composition imposes a curvature on the membrane, termed spontaneous curvature [46]. The concept of spontaneous curvature has been widely used to explain various membrane remodeling phenomena [47–50], specifically the observed shapes of RBCs from stomatocytes to discocytes and echinocytes [51–55]. Additionally, Kabaso *et al.* showed that the induced local spontaneous curvature due to the spatial attachment of spectrin filaments to the inner surface of the RBC lipid bilayer is a key mechanism that drives the inside-out membrane curling phenomena [56]. Despite differences in its molecular origin, spontaneous curvature is known to contribute to the membrane tension, in what is termed as spontaneous tension [57]. Dasgupta *et al.* have investigated the role of spontaneous tension in the malaria invasion by introducing an effective tension, including the contributions of both RBC membrane tension due to membrane inextensibility and spontaneous tension [4]. They found that for high effective tension, the transition between erythrocyte wrapping states is continuous, whereas, for low effective tension, the transition is associated with an energy barrier [4].
4. **Role of merozoite adhesion and line tension:** A critical step in malaria invasion is the adhesive interaction between the RBC membrane and the merozoite. For a full membrane-driven merozoite wrapping, the energy gained by merozoite adhesion to the RBC surface needs to overcome the energy cost due to membrane bending, membrane tension resistance, and cytoskeleton deformation [58, 59]. For instance, the absence of the Duffy antigen receptor for chemokines (DARC) on RBC surfaces significantly reduces merozoite adhesion to the membrane, which makes the Duffy-negative blood group resistant to malaria invasion [60]. Line tension at the boundary of the merozoite attachment site characterizes the discontinuity in membrane properties between the region adhering to the merozoite and the free membrane outside of the invagination domain [55]. Interfacial line tension at the merozoite boundary can create a nucleation barrier in the early stage of merozoite wrapping by increasing the energy required to form the initial membrane invagination [61]. Ignoring the effects of RBC cytoskeleton, Dasgupta et al. suggested that at low adhesion strength, interfacial forces impede the merozoite entry [4], but at high adhesion strength, these interfacial forces push the merozoite forward from partial wrapping to full wrapping [4].

Previous mathematical models have used the fluid membrane framework and interpreted the contribution of the spectrin network as an effective membrane tension [4, 6, 62, 63]. However, several studies have suggested that cytoskeletal remodeling and its posttranslational modifications can play crucial roles during merozoite invasion [16, 19, 38–40]. The RBC cytoskeleton is free to move in lateral directions. At the continuum level, the empirical constitutive equation based on thermodynamic invariants has been proposed by Evans and Skalak to describe the elastic energy of the cytoskeleton in the limit of small deformation [64]. This model has been extensively used, particularly in studies on red blood cell (RBC) membrane deformation in capillaries and echinocyte formation [32, 65–69]. Recently, Feng et al. proposed a microstructure-based elastic model that accounts for large cytoskeleton deformation and strain-hardening behavior at the spectrin level [70]. However, it remains unclear how the induced tension due to the coupled bilayer-cytoskeleton and the physical properties of the spectrin-actin network affect the progress of malaria invasion.

In this work, we sought to answer the following specific questions. How does RBC membrane tension including the effects of lipid bilayer incompressibility, spontaneous curvature, interfacial tension, and the cytoskeleton resistance against deformation, impact the morphological progression of parasite wrapping during malaria invasion? What are the roles of adhesion energy and interfacial line tension at the edge of merozoite in modulating these relationships? And finally, how do changes in the physical properties of RBCs alter the mechanical landscape of actomyosin forces required to complete invasion? To answer these questions, we used a general theoretical framework that incorporates the mechanics of a lipid bilayer with cytoskeleton deformation and membrane-merozoite interactions during the malaria invasion process. Our results show that the success of parasite invasion, as measured by the wrapping of the RBC membrane around the parasite, depends on the magnitude of the effective membrane tension of the RBC, which in turn depends on both the mechanics of the membrane and cytoskeleton.

## Model development

### Assumptions

**Fig 2:**
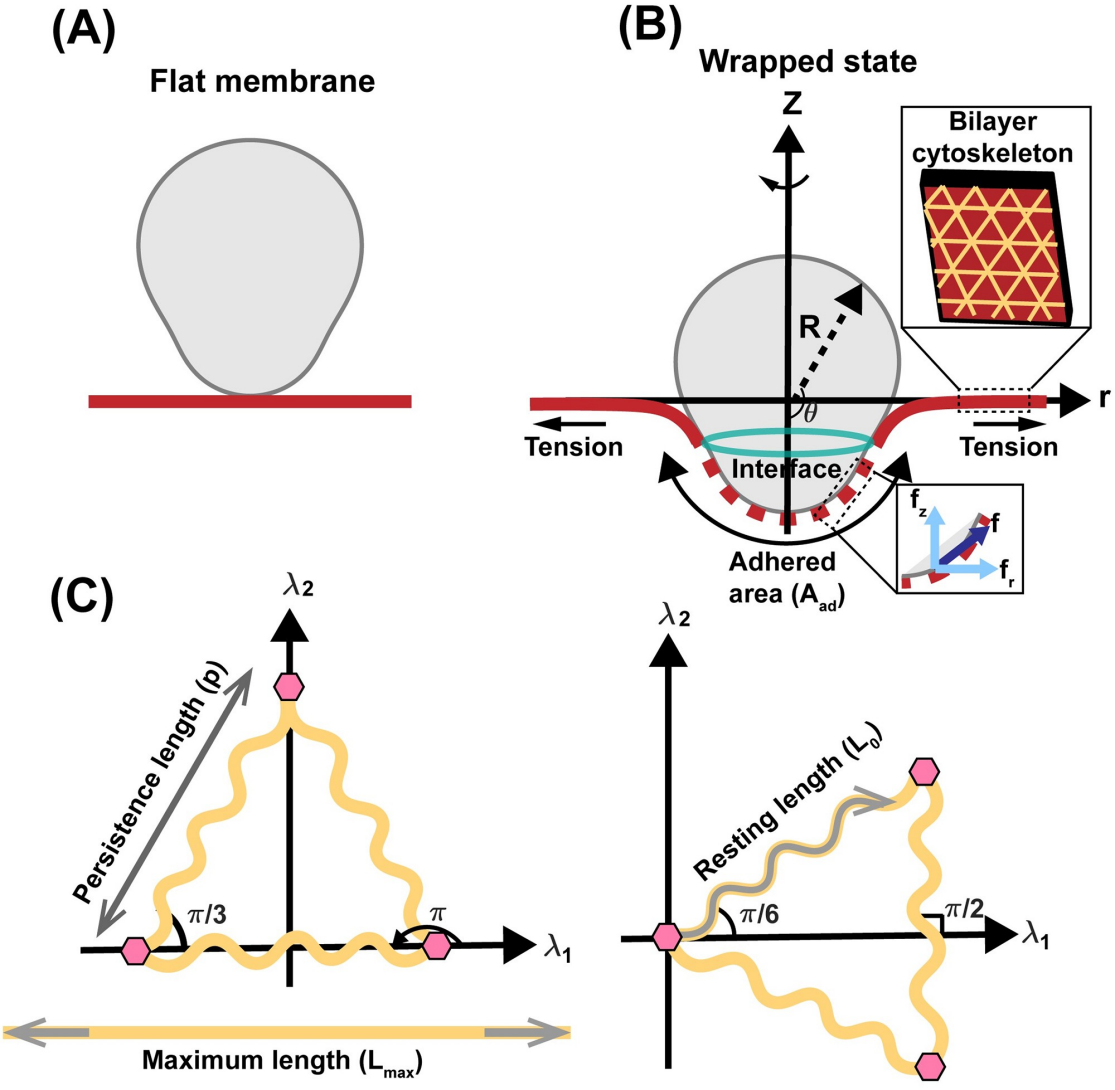
Schematic illustration of membrane wrapping and the RBC cytoskeleton. (A) Bilayer-cytoskeleton deforms from a flat circular patch to the membrane wrapping state. We model the lipid bilayer as a continuous thin elastic shell and the cytoskeleton as a hexagonal elastic network. (B) The axisymmetric parametrization of an idealized egg-shaped merozoite [4] in the wrapped state. The adhered part of the membrane is shown in dashed red, the free part of the membrane is shown in solid red, and their interface is shown by a green ring. R is the merozoite radius and *θ* is the wrapping angle. We assume that the actomyosin motors apply forces tangent to the surface (**f**) and the RBC membrane is under tension due to both lipid bilayer inextensibility and bilayer-cytoskeleton interactions. (C) The cytoskeleton network with two different orientations. λ_1_ and λ_2_ are the principal stretch directions. We model the spectrin filament as an elastic worm-like polymer with a persistence length of *p*, a maximum length of *L*_max_, and a resting length of *L*_0_ [70].

1. We model the RBC membrane as a two layer manifold with one layer for the lipid bilayer and the other for the cytoskeleton (Fig 2A). We assume that the system is at mechanical equilibrium at all times and neglect both fluctuations and inertia [71–73]. Analogous to the cup-like model [74], we assume that the free lipid bilayer and cytoskeleton outside of the parasite surface are almost flat and we only calculate the total free energy of the system on the adhered parasite area (Fig 2B) [4, 75].
2. We treat the lipid bilayer as a continuous thin elastic shell, assuming that the bilayer thickness is negligible compared to the radii of membrane curvature [46, 51]. We also assume that the lipid bilayer is incompressible and model the bending energy of the lipid bilayer using Helfrich–Canham energy, which depends on the local curvatures of the surface and bilayer properties [24, 46, 53, 76, 77].
3. We treat the cytoskeleton as a triangular elastic network with two different orientations and the network bonds (mediated by spectrin) that behave as an elastic worm-like polymer (Fig 2C) [78, 79]. This allows us to model the entropic free energy stored in the spectrin proteins using the Worm Like Chain (WLC) model [80, 81]. We also assume that the cytoskeleton convects with the bilayer, which imposes the areal inextensibility of the bilayer-cytoskeleton composite [82–86].
4. We model the contact energy between the merozoite surface and erythrocyte membrane with a contact potential, assuming that adhesive strength is homogeneous on the surface of the parasite [58, 59, 87]. Additionally, to accommodate the transition from the free membrane domain to the domain where the membrane adheres to the parasite, we consider the contribution of interfacial line tension at the edge of the adhering merozoite (Fig 2B) [4, 58, 88].
5. We assume that the movement of anchored myosin motors on the polymerized actin filaments inside the parasite pushes the RBC adhered area rearwards and propels the merozoite forward into the target cell (Fig 2B) [14]. We model the net effect of parasite motor forces as a work done on the RBC membrane and do not include the molecular details of the actomyosin assembly; these have been considered in [4, 34, 89].
6. For ease of computation, we assume that a flat circular patch of a lipid bilayer and relaxed cytoskeleton deformed to fit the merozoite contour in the adhesive region (Fig 2A) [66, 68]. We also assume that the shape of merozoite and the deformed bilayer/cytoskeleton composite are axisymmetric (Fig 2B) [4].

### Free energy of the system

The total energy of the system (*E*) is a sum of three terms: the energy associated with the bilayer-merozoite interactions (*E*_*b*_), the work done by the parasite actomyosin forces (*E*_*f*_), and the energy associated with the cytoskeleton deformation (*E*_*c*_)

$$\begin{array}{c} E=E_{b}-E_{f}+E_{c}. \end{array}$$ (1)

The bilayer-merozoite interactions energy includes the bending energy of the lipid bilayer, the work done against the lateral tension of the bilayer to pull excess membrane toward the parasite wrapping site, the adhesion energy due to merozoite attachment to the membrane surface, and the interfacial line tension at the edge of adhering merozoite, which is given as [4, 46, 87]

$$\begin{array}{c} E_{b}=\underset{\text{Bending}\;\text{energy}\;}{\underset{︸}{2\kappa \int_{A_{ad}}{\left(H\right)}^{2}da}}+\underset{\text{Work}\;\text{done}\;\text{against}\;\text{bilayer}\;\text{tension}}{\underset{︸}{\sigma_{\text{bilayer}}\Delta A}}-\underset{\text{Adhesion}\;\text{energy}\;}{\underset{︸}{\omega \int_{A_{ad}}da}}+\underset{\text{Line}\;\text{tension}}{\underset{︸}{\gamma \oint_{\partial l}dl}}, \end{array}$$ (2)

where *A*_*ad*_ is the surface area over the adhered parasite region, *H* is the membrane mean curvature, *σ*_bilayer_ is the lateral bilayer tension, Δ*A* is the excess area compared to a flat membrane, *κ* is the bending modulus of the lipid bilayer, *ω* is the adhesion energy per area, *γ* represents the strength of line tension, and the integral is over the interfacial line *dl*.

The work done on the membrane by applied forces by the parasite actomyosin motors is given by [34, 90]

$$\begin{array}{c} E_{f}=\int_{A_{ad}}f\cdot \left(r-r_{0}\right)da, \end{array}$$ (3)

where **f** is the applied force per unit area, **r** is the position vector in the current configuration, and **r**_0_ is the position vector in the reference frame. The energy density of the membrane cytoskeleton energy including the entropic energy stored in the spectrin proteins and the steric interactions between chain elements was recently derived in [70] building on the previous models presented in [79, 91]. We expand on the details in the supplementary material and provide the free energy density per unit area here for brevity.

For a 2-D triangle spectrin filament network with two different orientations, the spectrin persistence length *p*, the maximal spectrin chain length *L*_max_, the spectrin length of *L*_0_ in stress free state, the free energy density of the membrane skeleton (*W*_*c*_) can be written as (Fig 2C) [70, 92],

$$\begin{array}{c} \begin{array}{c} W_{c}=\underset{\text{Entropic}\;\text{energy}\;\text{of}\;\text{spectrin}\;\text{filaments}\;\text{orientations}}{\underset{︸}{\frac{2c_{\beta }}{3x_{0}^{2}}\sum_{\begin{array}{c} \phi =n\pi /6\\ n\in \{1,2,..6\} \end{array}}x_{0}^{2}(\lambda_{1}^{2}\text{cos}{\left(\phi \right)}^{2}+\lambda_{2}^{2}\text{sin}{\left(\phi \right)}^{2})\frac{3-2x_{0}\sqrt{\lambda_{1}^{2}\text{cos}{\left(\phi \right)}^{2}+\lambda_{2}^{2}\text{sin}{\left(\phi \right)}^{2}}}{1-x_{0}\sqrt{\lambda_{1}^{2}\text{cos}{\left(\phi \right)}^{2}+\lambda_{2}^{2}\text{sin}{\left(\phi \right)}^{2}}}}}+\\ \underset{\text{Steric}\;\text{interactions}}{\underset{︸}{c_{\beta }\frac{4x_{0}^{2}-9x_{0}+6}{{\left(1-x_{0}\right)}^{2}}}}, \end{array} \end{array}$$ (4)

where we define *x*_0_ = *L*_0_/*L*_max_, λ_1,2_ are the local principal stretches, and $c_{\beta }=\frac{\sqrt{3}k_{B}T}{4pL_{\text{max}}}$ (*k*_*B*_ is Boltzmann’s constant and *T* is the absolute temperature). The total energy of the skeleton can be obtained by the integral of the energy density over the adhered area to the parasite given by

$$\begin{array}{c} E_{c}=\int_{A_{ad}}W_{c}. \end{array}$$ (5)

Thus, the total energy of the membrane-cytoskeleton composite is given by the sum of Eqs 2, 3, and 5. We seek to calculate the change in the energy of the system from a locally flat state to a wrapped state (Fig 2A and 2B); this energy change is given by

$$\begin{array}{c} \begin{array}{c} \Delta E\left(\theta \right)=E_{\text{wrapped}}\left(\theta \right)-E_{\text{flat}}=\int_{A_{ad}}(2\kappa {\left(H-H_{0}\right)}^{2}-\omega -f\cdot \left(r-r_{0}\right)+W_{c})\;da\\ +\gamma \oint_{\partial l}\;dl+\sigma_{\text{bilayer}}\Delta A, \end{array} \end{array}$$ (6)

where *θ* is the wrapping angle and *H*_0_ is the parasite-induced spontaneous curvature on the bilayer surface.

### Numerical implementation

A key challenge in membrane biomechanics problems is energy minimization associated with mechanical equilibrium. Traditionally, we minimize the membrane energy using the principle of virtual work to obtain the shape of the membrane in response to induced curvatures and external forces [26, 93–98]. Here, we adopt an approach to solve the inverse problem. [99, 100]. Parameterizing the egg shape of the merozoite as (*X*^2^ + *Y*^2^ + *Z*^2^) = *R*_*a*_*X*^3^ + (*R*_*a*_ − *R*_*b*_)*X*(*Y*^2^ + *Z*^2^), with *R*_*a*_ = 1 *μ*m, *R*_*b*_ = 0.7 *μ*m [4], we fix the shape of the membrane adhered to the merozoite and minimize the energy (Eq 6) associated with mechanical equilibrium to find the degree of membrane wrapping θ* for any given set of membrane properties. This approach has the advantage of focusing on extent of wrapping without extensive computational overhead. Biologically relevant values for the parameters that have been used in the mathematical model are listed in Table 1.

**Table 1:** Parameters used in the model.

Parameter	Significance	Value	Ref(s)
*κ*	Bending rigidity	125 pN ⋅ nm	[22]
*σ*_bilayer_	Lipid bilayer tension	10^−4^—1 pN/nm	[22, 23, 34, 36, 101]
*ω*	Adhesion strength	10^−3^—1 pN/nm	[22, 102–106]
*γ*	Interfacial force	0—50 pN	[107, 108]
*H*_0_	Spontaneous curvature	0—0.1 nm^−1^	
*L*_0_	Resting length of spectrin	25—85 nm	[92, 109–112]
*L*_max_	Maximum length of spectrin	180—210 nm	[113–115]
*p*	Persistence length of spectrin	10—25 nm	[70]

## Results

### Successful malaria invasion is associated with an energy barrier controlled by effective membrane tension

**Fig 3:**
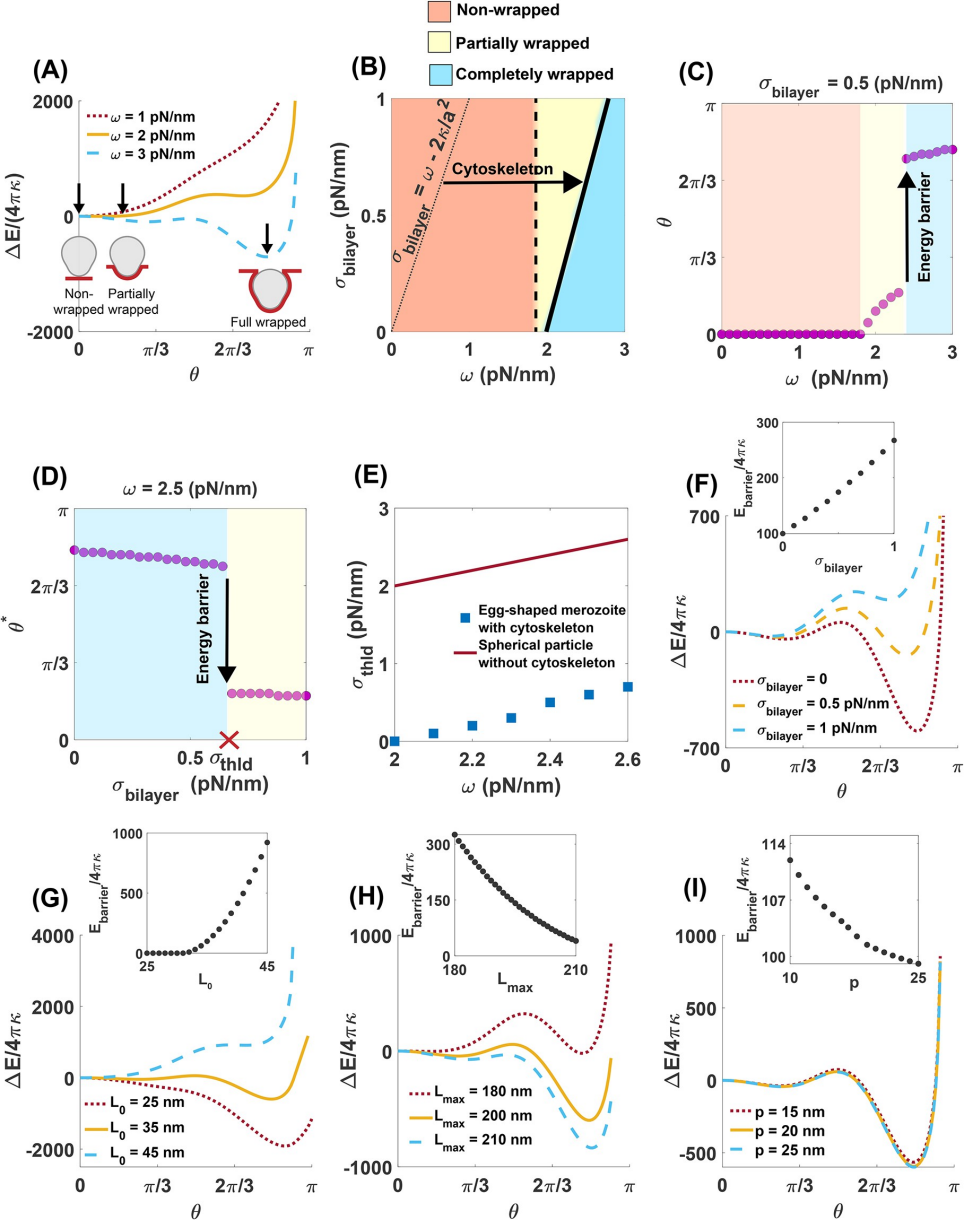
Complete merozoite wrapping by RBC membrane is associated with an energy barrier. (A) The change in the energy of the RBC bilayer/cytoskeleton composite as a function of wrapping angle (*θ*) for a fixed *σ*_bilayer_ = 0.5 pN/nm and three different magnitudes of adhesion strength. The change in the energy is minimized either for (*i*) a non-wrapped state (θ* = *θ*|_minimum energy_ = 0), or (*ii*) a partially wrapped state with a small wrapping fraction (0 < θ* < *π*/4), or (*iii*) a completely wrapped state (θ* > *π*/2). Arrows show the location of minimum energy. (B) Merozoite wrapping phase diagram for a range of lipid bilayer tension (*σ*_bilayer_) and adhesion strength (*ω*). The dashed and solid lines mark a continuous and a discontinuous transition, respectively. The dotted line represents the transition boundary below which a spherical particle is fully wrapped by the plasma membrane with no cytoskeletal effects (Eq S21). Wrapping angle at the minimized energy (θ*) as a function of (C) adhesion strength (*σ*_bilayer_ = 0.5 pN/nm) and (D) bilayer tension (*ω* = 2.5 pN/nm). The maximum bilayer tension required for a successful malaria invasion is marked as *σ*_thld_. (I) The bilayer tension threshold (*σ*_thld_) increases almost linearly as a function of adhesion strength. The height of the wrapping energy barrier as a function of (C) membrane tension, (D) the resting length of spectrin *L*_0_ (*p* = 25 nm and *L*_*max*_ = 200 nm), (E) the maximum length of spectrin *L*_max_ (*p* = 25 nm and *L*_0_ = 35 nm), and (F) the persistence length of spectrin *p* (*L*_0_ = 35 nm and *L*_*max*_ = 200 nm) with *ω* = 2.5 pN/nm. In panels A-F, *p* = 25 nm, *L*_0_ = 35 nm, and *L*_*max*_ = 200 nm.

Motivated by a recent experimental observation that high RBC membrane tension can inhibit malaria invasion [22], we asked how does the interplay between the strength of merozoite adhesion and membrane tension due to lipid inextensibility and the shear elasticity of the cytoskeleton affect the degree of parasite wrapping around the RBC membrane? To answer this question, we calculated the change in the energy of bilayer/cytoskeleton composite (Eq 6) as a function of wrapping angle (*θ*) for a fixed *σ*_bilayer_ = 0.5 pN/nm and three different magnitudes of adhesion strength (Fig 3A). To answer this question, we calculated the change in the energy of bilayer/cytoskeleton composite (Eq 6) as a function of wrapping angle (*θ*) for two different magnitudes of bilayer tension and adhesion strengths (Fig 3A). Here, we set *p* = 25 nm, *L*_0_ = 35 nm, and *L*_max_ = 200 nm and there is no spontaneous curvature (*H*_0_ = 0) and no interfacial force (*γ* = 0). We find that depending on the magnitude of adhesion strength, there are three states which minimize the change in the energy; (*i*) a non-wrapped state (θ* = 0), (*ii*) a partially wrapped state with a small wrapping fraction (0 < θ* < *π*/4), and (*iii*) a completely wrapped state (θ* > *π*/2) (Fig 3A). As can be seen in Fig 3A, the partially wrapped state is separated from the completely wrapped state by an energy barrier. Additionally, we found that the total energy of the bilayer/cytoskeleton composite diverges toward ∞ as *θ* → *π* (Fig 3A). This is because the first principle stretch (λ_1_) and the energy associated with the cytoskeleton resistance diverges for large deformations (Eq S17). Thus, we conclude that the complete merozoite entry into the RBCs requires a local disassembly of the cytoskeleton.

In Fig 3B, we plotted the merozoite wrapping phase diagram for a range of bilayer tension (*σ*_bilayer_) and adhesion strength (*ω*). The orange region denotes a non-wrapped state, the yellow region represents a partially wrapped state, and the blue region indicates a state where a merozoite is completely wrapped by the RBC membrane (Fig 3B). We marked the continuous transition between the non-wrapped state (orange region) and the partially wrapped state (yellow region) and the discontinuous transition between the partially wrapped state and the completely wrapped state (blue region) by dashed and solid lines, respectively (Fig 3B). In the case of a spherical particle wrapping with no cytoskeletal effects, the sphere is fully wrapped by membrane when *σ*_bilayer_ < *ω* − 2*κ*/*a*^2^ (shown as a dotted line in Fig 3B). Here, *a* is the radius of the sphere with the same surface area as the egg-shaped parasite (Eq S21) [59]. As expected, the cytoskeletal resistance against deformation shifts the transition to successful invasion toward the higher adhesion strengths (arrow in Fig 3B), implying that larger adhesive forces are required for successful entry into an RBC. In Fig 3C and 3D, we show the discontinuous transition between partially wrapped states (failed invasion) and completely wrapped states (successful invasion) with increasing the magnitude of adhesion strength and bilayer tension, respectively.

The discontinuous transition between the partially and completely wrapped states is in agreement with the proposed concept of membrane tension threshold for successful malaria invasion in a recent study by Kariuki et al. [22]. Additionally, Dasgupta et al. [4] and other studies have shown the existence of energy barriers in merozoite wrapping by RBC membrane and generally in the wrapping of nanoparticles by cellular membranes [59, 116]. We marked the maximum tension above which the invasion is impaired as *σ*_thld_ (Fig 3D). Kariuki et al. [22] suggested a tension threshold in order of 10^−4^ pN/nm to limit malaria invasion. However, based on our results, *σ*_thld_ is almost linearly proportional to the adhesion strength; lower *σ*_thld_ is required when the parasite adhesion strength is smaller (Fig 3E). The linear relationship between the tension threshold and adhesion strength was expected from the analytical expression, *σ*_thld/no cytoskeleton_ ∼ *ω* (Eq S21). However, our results show that the resistance of the cytoskeleton against deformation shifts the tension threshold to significantly lower values (Fig 3E).

To investigate the contribution of the induced tension by the lipid bilayer and the cytoskeleton layer on the energy barrier associated with the complete merozite wrapping, we plotted the change in the energy of the system and the height of the energy barrier for different bilayer tension (*σ*_bilayer_), resting length of the spectrin (*L*_0_), maximum length of spectrin (*L*_*max*_), and persistence length of spectrin (*p*), with a fixed adhesion strength, *ω* = 2.5 pN/nm (Fig 3F-3I). We found that with a cytoskeleton (*p* = 25 nm, *L*_0_ = 35 nm, and *L*_*max*_ = 200 nm), the height of the energy barrier becomes 3 times larger, reaching *E*_barrier_ ∼ 3 × 10^4^*k*_*B*_*T*, as the bilayer tension increases from *σ*_bilayer_ = 0 to *σ*_bilayer_ = 1 pN/nm.

However, without a cytoskeleton, there is no energy barrier to completely wrap the merozoite, even at a high bilayer tension of *σ*_bilayer_ = 1 pN/nm (Fig A in S1 Text). Based on our results, the resistance of the cytoskeleton to shear deformation (*σ*_bilayer_ = 0) can create a large energy barrier for wrapping the merozoite. Particularly, an increase in the resting length of the spectrin can raise the energy barrier to > 10^5^*k*_*B*_*T* (Fig 3G). However, changes in the persistence length of spectrin have a negligible effect on the height of the energy barrier (Fig 3I). Overall, our model predicts that RBC membrane tension sets an energy barrier for a complete merozoite wrapping, while the minimum tension to impede the malaria invasion depends linearly on the strength of merozoite adhesion to the RBC membrane.

### Physical properties of RBC cytoskeleton control the shear elasticity of the membrane and the efficiency of malaria invasion

We next asked how do the physical properties of the spectrin network alter the resistance of the membrane against parasite wrapping? In this study, we assumed that the membrane cytoskeleton is areally incompressible and the spectrin network can only move in lateral directions (shear deformation). The resistance to the shear deformation is represented by the shear modulus *μ*. Initially, it has been proposed that the shear modulus of the RBC cytoskeleton is constant and is in the order of *μ* ∼ 2.5 pN/*μ*m [117–119]. However, recent studies have suggested that similar to the nonlinear response of biopolymers, the shear modulus magnitude of the spectrin network in the RBC cytoskeleton depends on the extension ratios and exhibits a strain hardening behavior in large deformations [69, 70]. For example, using a WLC model, Feng et al. [70] derived the shear modulus of an incompressible RBC cytoskeleton as a function of principal stretches and the physical properties of the cytoskeleton (Eq. 19 in [70]).

**Fig 4:**
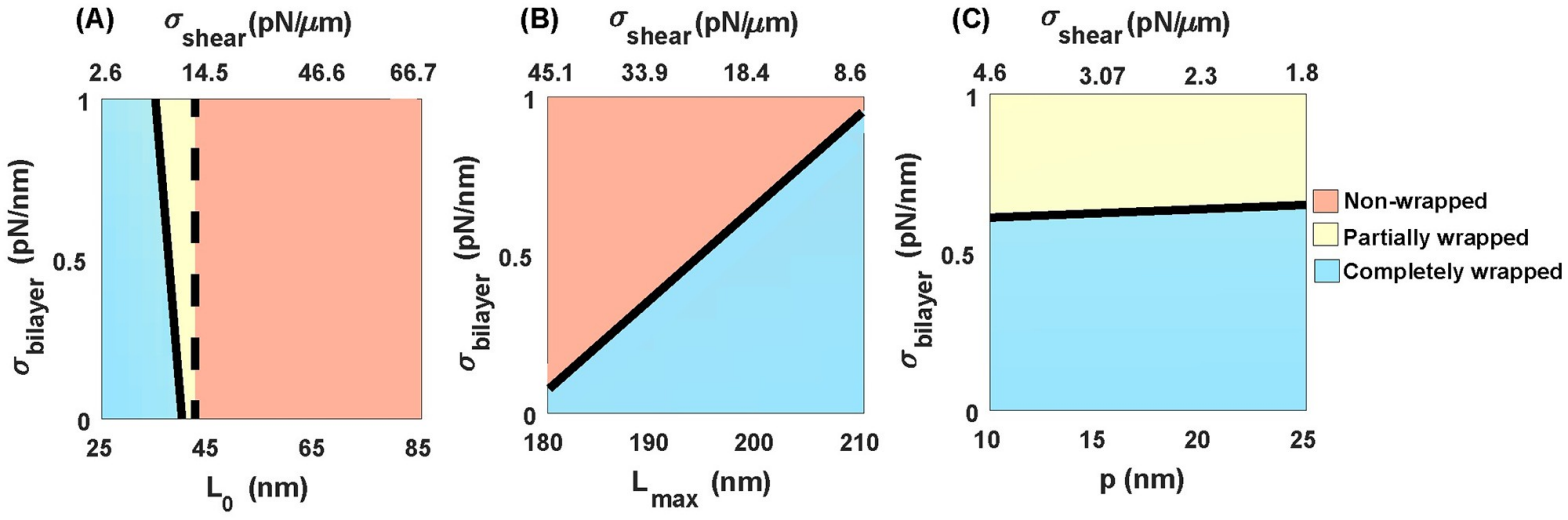
Significance of RBC cytoskeleton for inhibiting parasite invasion, *ω* = 2.5 pN/nm. Merozoite wrapping phase diagram for a range of bilayer tension and (A) the resting length of spectrin *L*_0_ (*p* = 25 nm and *L*_*max*_ = 200 nm), (B) the maximum length of spectrin *L*_max_ (*p* = 25 nm and *L*_0_ = 35 nm), and (C) the persistence length of spectrin *p* (*L*_0_ = 35 nm and *L*_*max*_ = 200 nm). In all panels, the colors show the same wrapping states as Fig 3. The dashed and solid lines represent the continuous and discontinuous transition between the states, respectively. Shear tension (*σ*_shear_) is defined as the average shear modulus of the cytoskeleton (average of *μ* in Eq. 19 in [70]).

To understand how the physical properties of the cytoskeleton affect the resistance of the RBC membrane against the malaria invasion, we fixed *H*_0_ = 0, *γ* = 0, *ω* = 2.5 pN/nm, and plotted the wrapping phase diagrams for the egg-shaped merozoite for a range of bilayer tension and natural length of spectrin (*L*_0_), the maximum length of spectrin (*L*_max_), and persistence length of spectrin (*p*) (Fig 4A-4C). We defined the average of the shear modulus of the cytoskeleton (average of *μ* in Eq. 19 in [70]) as a shear tension (*σ*_shear_). The value of the shear tension is given at the top of each panel in Fig 4A-4C. Based on our results, the merozoite wrapping process is inhibited by increasing the natural length of spectrin from *L*_0_ = 25 nm to *L*_0_ = 85 nm (Fig 4A). This is because a larger *L*_0_ results in a higher shear tension which makes the cytoskeletal layer more rigid against deformation (Fig 4A). In contrast to the natural length of spectrin, we found that the shear tension of the cytoskeleton decreases with an increase in the maximum or persistence length of spectrin (Fig 4B and 4C). This decreases in the magnitude of shear tension facilitates the complete merozoite wrapping transition (Fig 4B and 4C). It should be mentioned in all panels of Fig 4, the transition between the completely and partially wrapped states is discontinuous (shown by a solid line), but the transition between the partial and non-wrapped states is continuous (shown by a dashed line) (see Fig C in S1 Text). From these results, we can conclude that the physical properties of the cytoskeleton play key roles in specifying the magnitude of shear tension and, ultimately, the RBC resistance against malaria invasion.

### Spontaneous tension can impede malaria invasion

**Fig 5:**
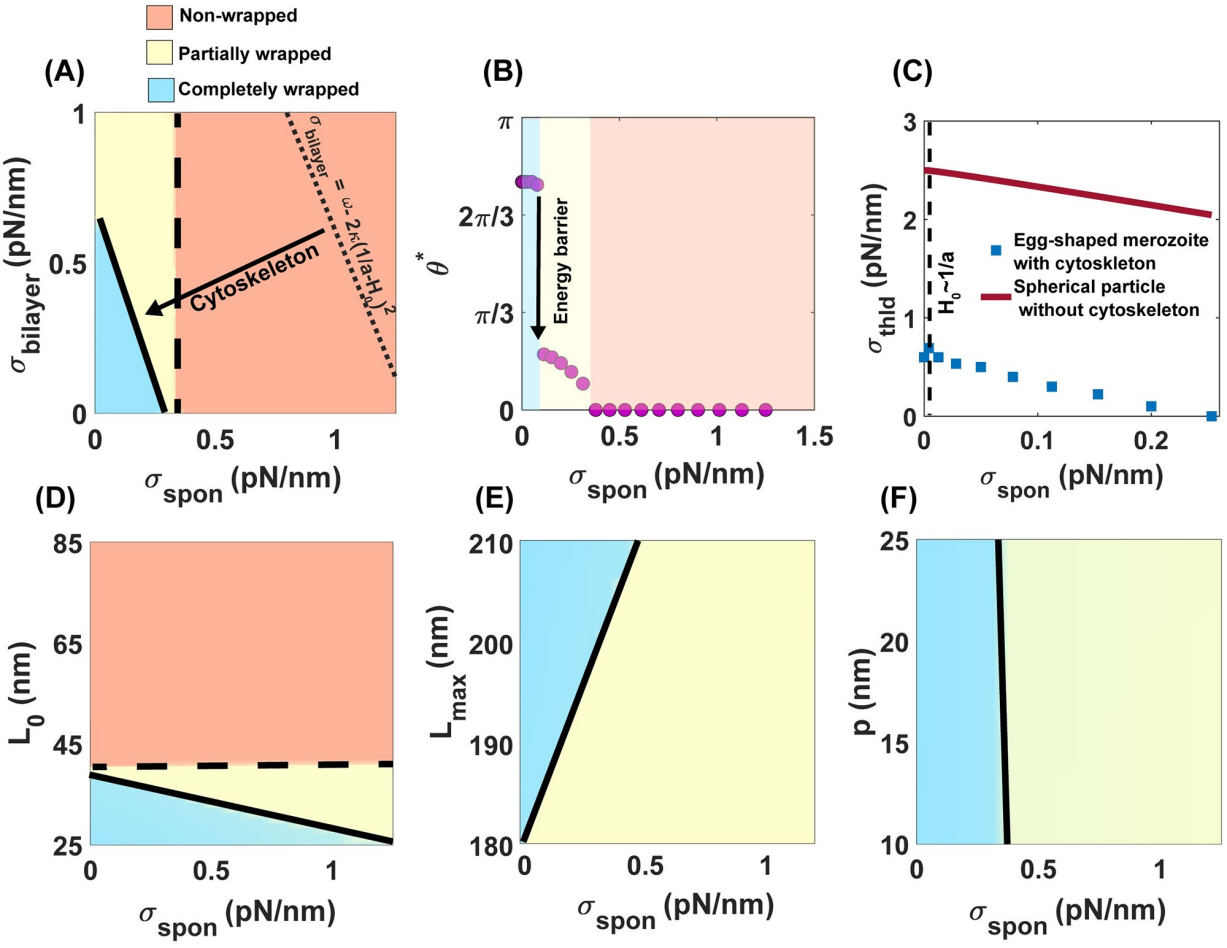
Large spontaneous tension results in malaria invasion resistance (*ω* = 2.5 pN/nm). (A) Merozoite wrapping phase diagram for a range of bilayer tension (*σ*_bilayer_) and induced spontaneous tension ($\sigma_{\text{spon}}=\kappa H_{0}^{2}$) due to the local attachment of merozoites on the RBC surface. The colors indicate the same wrapping states as Fig 3 and the solid line demonstrates the discontinuous transition between states. The dotted line represents the analytical approximation for wrapping of a spherical particle with no cytoskeletal effects (Eq S22). (B) With increasing the magnitude of induced spontaneous tension, there is an energy barrier that separates the partially and completely wrapped states (*σ*_bilayer_ = 0.5 pN/nm). (C) The nonmonotonic behavior of membrane tension threshold (*σ*_thld_) with increasing the induced spontaneous tension. In panels A-C, *p* = 25 nm, *L*_0_ = 35 nm, *L*_max_ = 200 nm. Merozoite wrapping phase diagram for a range of induced spontaneous tension (*σ*_bilayer_ = 0) and (D) the resting length of spectrin *L*_0_ (*p* = 25 nm and *L*_*max*_ = 200 nm), (E) the maximum length of spectrin *L*_max_ (*p* = 25 nm and *L*_0_ = 35 nm), and (F) the persistence length of spectrin *p* (*L*_0_ = 35 nm and *L*_*max*_ = 200 nm).

It has been suggested that any asymmetry between the leaflets of the lipid bilayer or surrounding environment can induce a relatively large spontaneous tension in order of 1 pN/nm [57]. We next investigated how this induced spontaneous tension can change the efficiency of malaria invasion. Based on the analytical approximations for the membrane wrapping of a spherical particle with no cytoskeletal effects, the successful invasion occurs when *σ*_bilayer_ < *ω* − 2*κ*(1/*a* − *H*_0_)^2^ (below dotted line in Fig 5A, Eq S22). This suggests when the spontaneous curvature is smaller than the curvature of the merozoite (*H*_0_ < 1/*a*), the induced spontaneous tension facilitates merozoite wrapping. However, a large spontaneous curvature (*H*_0_ > 1/*a*) increases the membrane resistance to invasion. To investigate the engulfment of an egg-shaped merozoite by the RBC membrane, we set the physical properties of the membrane cytoskeleton as Fig 3 (*p* = 25 nm, *L*_0_ = 35 nm, *L*_max_ = 200 nm) and plotted the wrapping phase diagram for a range of bilayer tension (*σ*_bilayer_) and induced spontaneous tension ($\sigma_{\text{spon}}=\kappa H_{0}^{2}$, *κ* is fixed and *H*_0_ varies) at a fixed adhesion strength, *ω* = 2.5 pN/nm (Fig 5A).

Considering the energy contribution due to the elastic deformation of the membrane cytoskeleton (Eq 5), we found that a failed malaria invasion for an egg-shaped merozoite shifts toward the smaller spontaneous tension, *σ*_spon_ < 0.5 pN/nm (arrow in Fig 5A). To better visualize the effect of spontaneous tension on the merozoite wrapping transition, we plotted the wrapping angle (θ*) as a function of spontaneous tension at *σ*_bilayer_ = 0.5 pN/nm (Fig 5B). As the induced spontaneous tension increases, we observed a discontinuous transition from a completely wrapped state to a partially wrapped state and then a continuous transition from a partially wrapped state to a non-wrapped state (Fig 5B). Based on our results, *σ*_thld_ is a nonmonotonic function of spontaneous tension; as spontaneous tension increases, *σ*_thld_ increases and then decreases again (Fig 5C). This is consistent with the analytical approximation for wrapping a spherical particle with no cytoskeletal effects. We also plotted the wrapping phase diagram for a range of cytoskeleton properties and induced spontaneous tension, *σ*_bilayer_ = 0 (Fig 5D-5F). Our findings indicate that for a cytoskeleton with a short resting length and a long maximum length, a substantial induced spontaneous tension (*σ*_spon_ > 0.5 pN/nm) is required to impede malaria invasion (Fig 5D and 5E). Thus, we predict that a large spontaneous tension can act as a protective mechanism against malaria invasion.

### Interfacial tension creates a nucleation barrier in merozoite wrapping

How do interfacial forces between the membrane at the site of invasion and the free membrane outside of adhered area influence the parasite wrapping behavior? Based on analytical approximation, the effects of interfacial forces on the wrapping process of a spherical particle with no cytoskeletal layer can be classified into three regimes (Eq S23). (*i*) Interfacial forces create a large energy barrier (“nucleation barrier”) such that the particle does not attach to the membrane (θ* < 0) [58, 61, 120]. (*ii*) Interfacial forces create an energy barrier that impedes the full envelopment of a partially wrapped particle by the membrane (θ* < *π*/2) (Eq S23a). (*iii*) Interfacial forces facilitate the encapsulation process when the membrane wrapping passes the equator of the sphere (θ* > *π*/2) (Eq S23b) [61, 120]. Physically, the net effects of interfacial forces or line tension (*γ*) between two boundaries with different properties can be represented by an interfacial tension defined as *σ*_inter_ = *γ*^2^/*κ*. To understand how interfacial tension impacts the entry of an egg-shaped merozoite into an RBC with a cytoskeleton layer, we plotted the wrapping phase diagram for a range of bilayer tension (*σ*_bilayer_) and interfacial tension (*γ*^2^/*κ*, *κ* is fixed and *γ* varies). Here, we considered a wide range of line tension (0 < *γ* < 50 pN) to mimic line tension at lipid domain boundaries and line tension due to protein phase separation [107, 108].

**Fig 6:**
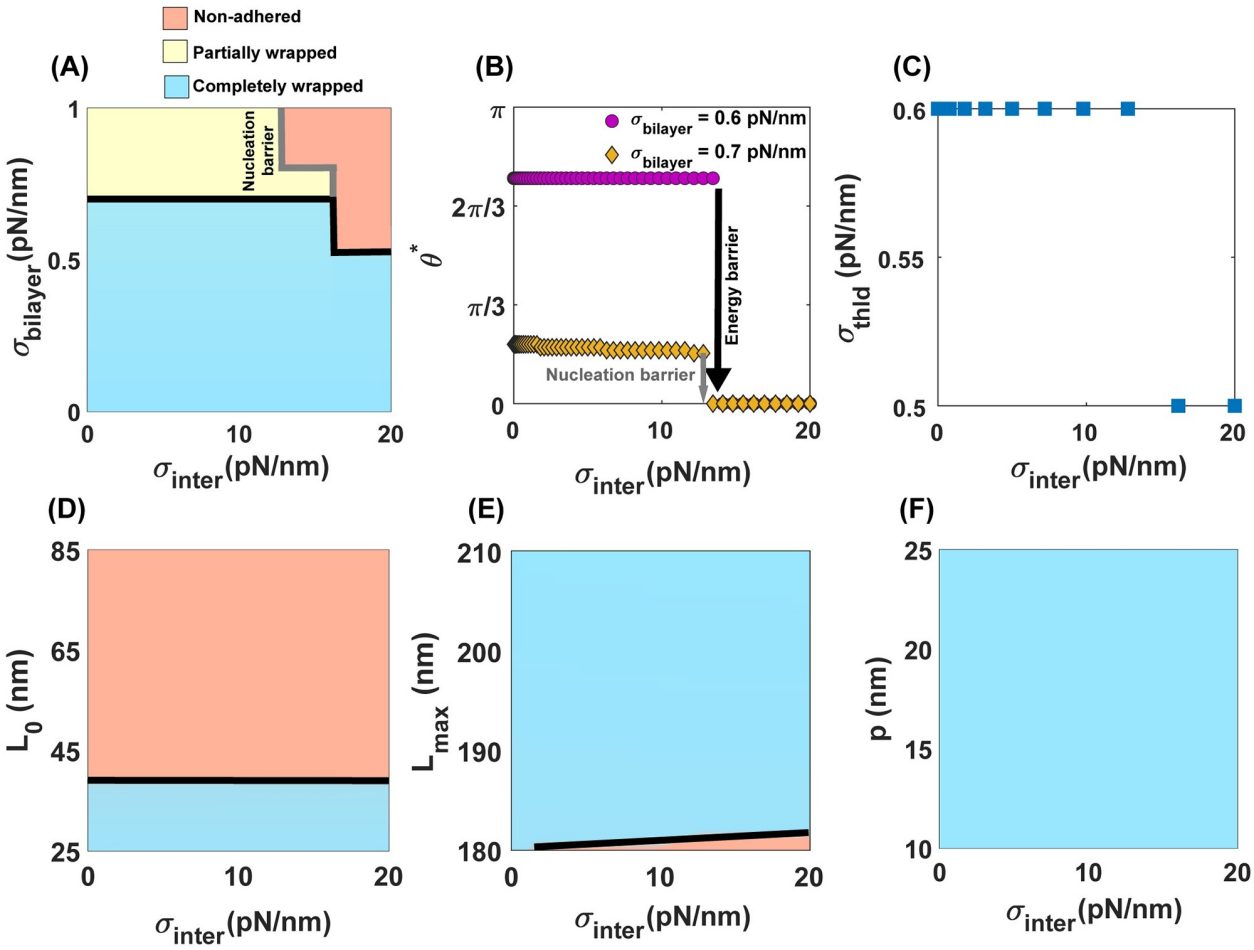
Interfacial tension plays an important role in regulating the success or failure of malaria invasion (*ω* = 2.5 pN/nm). (A) Merozoite wrapping phase diagram for a range of bilayer tension (*σ*_bilayer_) and induced interfacial tension (*σ*_inter_ = *γ*^2^/*κ*). Yellow and blue colors indicate the same wrapping states as Fig 3, while the orange color represents a non-adhered state (θ* < 0). The solid lines illustrate the discontinuous transitions associated with energy barriers between different states. We marked the nucleation barrier between the partially wrapped state and the non-adhered region by a solid gray line. (B) θ* as a function of induced interfacial tension for two different bilayer tensions. With increasing the magnitude of induced interfacial tension, there is a discontinuous transition from completely and partially wrapped states to the non-adhered state. (C) A switch-like behavior in the tension threshold (*σ*_thld_) with increasing the magnitude of interfacial tension. In panels A-C, *p* = 25 nm, *L*_0_ = 35 nm, *L*_*max*_ = 200 nm. Merozoite wrapping phase diagram for a range of induced interfacial tension (*σ*_bilayer_ = 0) and (D) the resting length of spectrin *L*_0_ (*p* = 25 nm and *L*_*max*_ = 200 nm), (E) the maximum length of spectrin *L*_max_ (*p* = 25 nm and *L*_0_ = 35 nm), and (F) the persistence length of spectrin *p* (*L*_0_ = 35 nm and *L*_*max*_ = 200 nm).

We can identify three different tension regimes in Fig 6A. At low bilayer tension (*σ*_bilayer_ < 0.5 pN/nm), independent of the magnitude of the interfacial tension, the particle is fully wrapped by the membrane. At intermediate bilayer tension (0.5 < *σ*_bilayer_ < 0.7 pN/nm), we observed a distinct contrast with the membrane wrapping of a spherical particle with no cytoskeleton layer (Eq S23b), wherein an increase in interfacial tension results in a discontinuous transition from a completely wrapped state to a non-adhesive state (Fig 6A and 6B). This discrepancy could arise from the combined effects of the merozoite’s egg-like shape and the substantial energy associated with the cytoskeleton deformation in the fully wrapped state (Fig 3A). In particular, for membrane wrapping of an egg-shaped merozoite with no cytoskeleton layer, we found that, depending on the combinations of the bilayer tension and adhesion strength, increasing the magnitude of interfacial tension leads to the transition of a wrapping state with θ* > *π*/2 to either a fully wrapped state of θ* = *π* (Fig B in S1 Text) or a non-adhered state of θ* = 0 (Fig B in S1 Text), which is in agreement with the previous study [4]. Finally, in the case of high bilayer tension (*σ*_bilayer_ > 0.7 pN/nm), we found that similar to the membrane wrapping of the spherical particle and the egg-shape merozoite with no cytoskeleton layer (Fig B in S1 Text), interfacial tension creates a nucleation barrier (shown by a solid gray line in Fig 6A) which separates a partially wrapped state from a non-adhered region (Fig 6A and 6B).

In Fig 6C, we plotted *σ*_thld_ as a function of interfacial tension. Interestingly, we observed a switch-like behavior in *σ*_thld_, wherein *σ*_thld_ sharply drops from 0.6 pN/nm to 0.5 pN/nm with increasing interfacial tension (Fig 6C). We also plotted the wrapping phase diagram for a range of cytoskeleton properties and induced interfacial tension, *σ*_bilayer_ = 0 (Fig 6D-6F). Interestingly, we found that the induced interfacial tension does not change the wrapping state, except in a narrow region with ∼ *L*_*max*_ = 180 nm depicted in Fig 6E. In this region, there is a discontinuous transition from a completely wrapped state to a non-wrapped state with the increasing magnitude of induced interfacial tension. (Fig 6E). Thus, these results suggest that interfacial tension coupled with bilayer tension can inhibit malaria invasion by creating two energy barriers; (*1*) a nucleation barrier that impedes the merozoite attachment to the RBC membrane and (*2*) a wrapping energy barrier that hinders the full merozoite entry.

### Biophysical properties of RBCs alter the magnitude of actomyosin forces required for a successful malaria invasion

**Fig 7:**
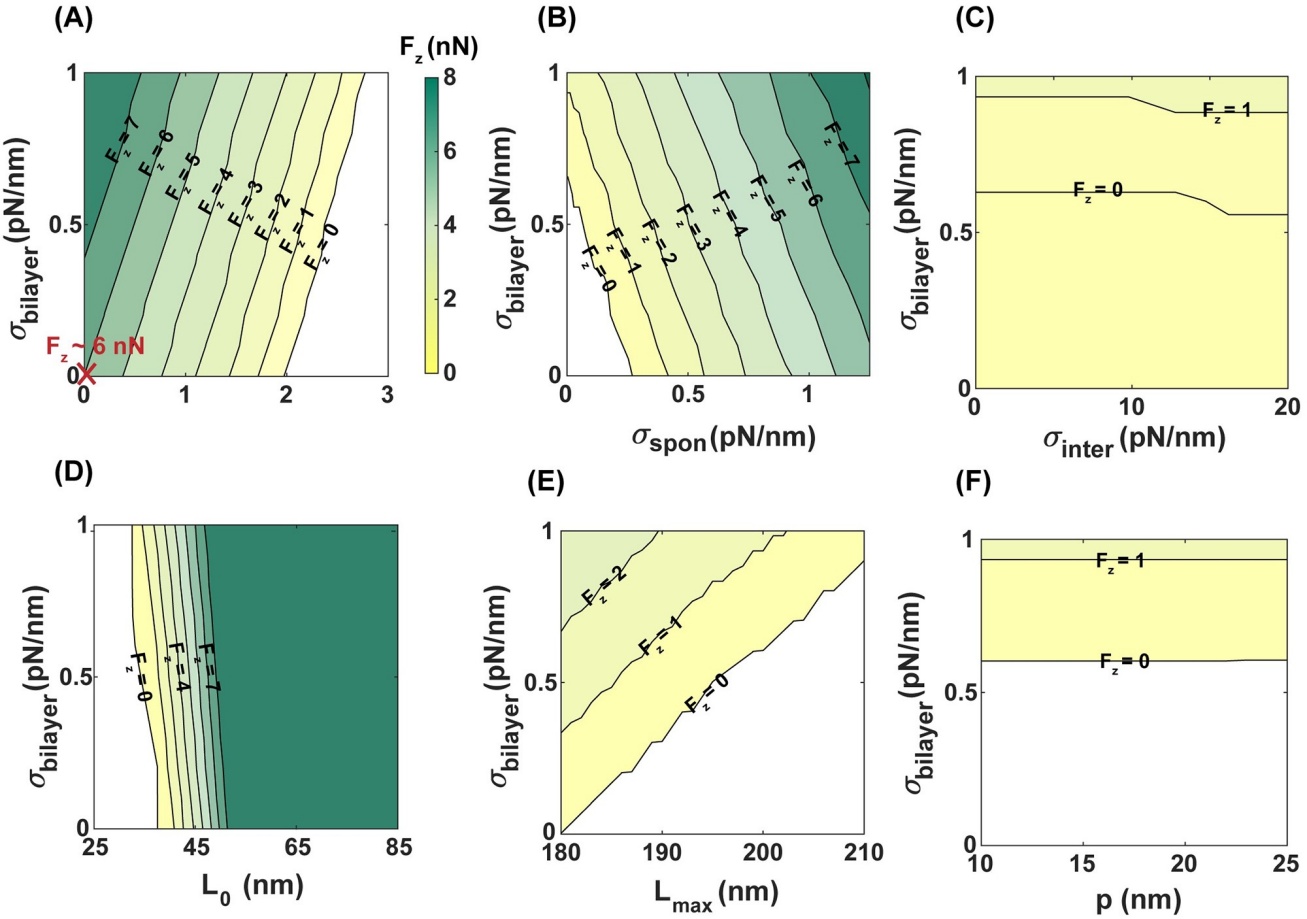
The magnitude of actomyosin forces required for a complete invasion depends on the biophysical properties of RBC membrane. Contour plots of the required actomyosin forces for a complete membrane wrapping for a range of bilayer tension and (A) the adhesion strength, (B) the spontaneous tension, (C) interfacial tension, (D) the resting length of cytoskeleton *L*_0_, (E) the maximum length of cytoskeleton *L*_*max*_, and (F) the persistence length of cytoskeleton *p*. In panels A-C, the physical properties of the cytoskeleton are set as *p* = 25 nm, *L*_0_ = 35 nm, and *L*_*max*_ = 200 nm and the results in panels B-F, are obtained for *ω* = 2.5 pN/nm. Also, in panels D-F, we set *H*_0_ = 0 and *γ* = 0 to focus on the cytoskeleton effects. The marked point **X** in panel A shows the minimum axial force (*F*_*z*_ ∼ 6 nN) required for successful invasion in a bilayer/cytoskeleton composite with a tensionless bilayer, no adhesion energy, no spontaneous curvature, and no line tension.

While the parasite’s motor forces are known as the primary driving mechanism for malaria invasion [11–14], we next asked whether the magnitude of motor-driven forces varies based on the biophysical properties of the host RBC membrane. To answer this question, we first estimated the minimum axial forces (*F*_*z*_) required for a full envelopment of a spherical particle with no cytoskeleton layer (Eq S26). Based on our analytical approximation, *F*_*z*_ is linearly proportional to the lipid bilayer bending rigidity (*κ*), bilayer tension (*σ*), adhesion strength (*ω*), and line tension (*γ*), and it varies as a quadratic function of spontaneous curvature (*H*_0_) (Eq S26). For example, in the case of a tensionless bilayer, with no adhesion energy, no spontaneous curvature, and no line tension, a minimum axial force of *F*_*z*_ ∼ 3 pN is required for a full wrapping of a spherical particle (Eq. S26), which is of the order of the reported actomyosin forces needed for a complete merozoite invasion by Dasgupta et al. [4]. Taking into account the energy contribution of cytoskeleton deformation, we numerically calculated the minimum axial forces required for a successful invasion by an egg-shaped merozoite, considering a range of lipid bilayer and cytoskeleton properties (Fig 7A-7F).

Consistent with the analytical approximations for a membrane wrapping of a spherical particle with no cytoskeleton layer, we observed a linear increase in the magnitude of the axial force (*F*_*z*_) with respect to bilayer tension and spontaneous tension, and a linear decrease as adhesion strength increases (Fig 7A-7B and Fig D in S1 Text). Moreover, our numerical results show that *F*_*z*_ undergoes a switch-like transition from a smaller to a larger value in response to increasing interfacial tension (Fig 7C and Fig D in S1 Text). Based on our results, the magnitude of axial forces required for complete merozoite entry significantly increases when taking into account the shear energy associated with cytoskeleton deformation (Fig 7 and Fig D in S1 Text). For example, considering the case of a bilayer/cytoskeleton composite with a tensionless bilayer, with no adhesion energy, no spontaneous curvature, and no line tension, a minimum axial force of *F*_*z*_ ∼ 6 nN is required for a successful invasion (Fig 7A). This is almost three orders of magnitudes larger than the case with no cytoskeletal layer [4].

In Fig 7D-7F, we showed the effect of the spectrin length scales on the degree of forces required to facilitate parasite transitions to a completed invasion. As expected from Fig 4, larger axial forces are needed for a cytoskeleton network with longer spectrin filaments in the resting condition (Fig 7D) or shorter spectrin filaments in the maximum stretched state (Fig 7E) and in the persistence state (Fig 7F). Based on our results, the natural length scale of spectrin (*L*_0_) has a considerable effect –compared to the other length scales of spectrin (*L*_*max*_ and *p*)– on the degree of motor forces required for a successful invasion (Fig 7D-7F). For example, for fixed bilayer properties and *L*_0_ = 25 nm, the merozoite is completely wrapped with no need for extra forces (*F*_*z*_ = 0), while at *L*_0_ = 50 nm, a minimum axial force of *F*_*z*_ ∼ 8 nN is required to push the parasite into the RBC (Fig 7D). This is because the shear tension of the cytoskeleton strongly depends on the natural length scale of the spectrin filaments. Overall, these results indicate that the biophysical properties of the RBC lipid bilayer and cytoskeleton layer adjust the degree of the motor forces required for a complete merozoite invasion. Particularly, our mechanical model predicts that large actomyosin forces (*F*_*z*_ ∼ O (1 nN)) are needed to drive the merozoite forward into the RBC, considering the resistance of the cytoskeletal layer against deformation.

### Conclusions and discussion

During the blood stage of malaria infection, thousands of merozoites, which are the smallest egg-shaped parasites with a typical size of 1–2 *μ*m, invade healthy RBCs and asexually reproduce inside them. The invasion process was initially assumed to be solely driven by the parasite actomyosin motor forces. However, recent experiments have shown that the biophysical properties of the RBC membrane, particularly the tension of the RBC membrane, also play an important role in controlling the malaria invasion [15, 22, 121]. The RBC membrane is a two layer manifold composed of an incompressible lipid bilayer and elastic spectrin-actin network. Previous theoretical studies for malaria invasion have not considered the active role of the RBC cytoskeleton and focused on the effect of induced tension within the lipid bilayer in regulating malaria invasion [4, 62].

Evans and Skalak initially proposed the empirical constitute elastic energy of the spectrin-actin network at the continuum level, treating the cytoskeleton as an isotropic hyperelastic material with constant shear and stretch moduli [117]. This classical model was able to explain the RBC deformability in capillaries and echinocyte formation with genetic defects [32, 64–69]. Subsequent extensions of this model include consideration of molecular details of the spectrin network [79, 91, 122, 123]. For example, for small deformations, Dao et al. calculated shear and area moduli of the cytoskeletal layer based on the virial stress at the spectrin level [124]. Additionally, Hendrickson et al. extended the mechanics of lipid bilayer with a conforming cytoskeletal layer [82]. They modeled the lipid bilayer as a nematic liquid crystal and assumed that the cytoskeleton is tethered to it by a so-called connector field [82]. Recently, Feng et al. derived an analytical hyperelastic constitutive model for the RBC cytoskeleton using the macroscopic behavior of spectrin filaments as a WLC [70]. Their proposed model accounts for the distribution of orientations and natural lengths of spectrin, representing the strain-hardening behavior of the RBC membrane observed in experiments [70].

**Fig 8:**
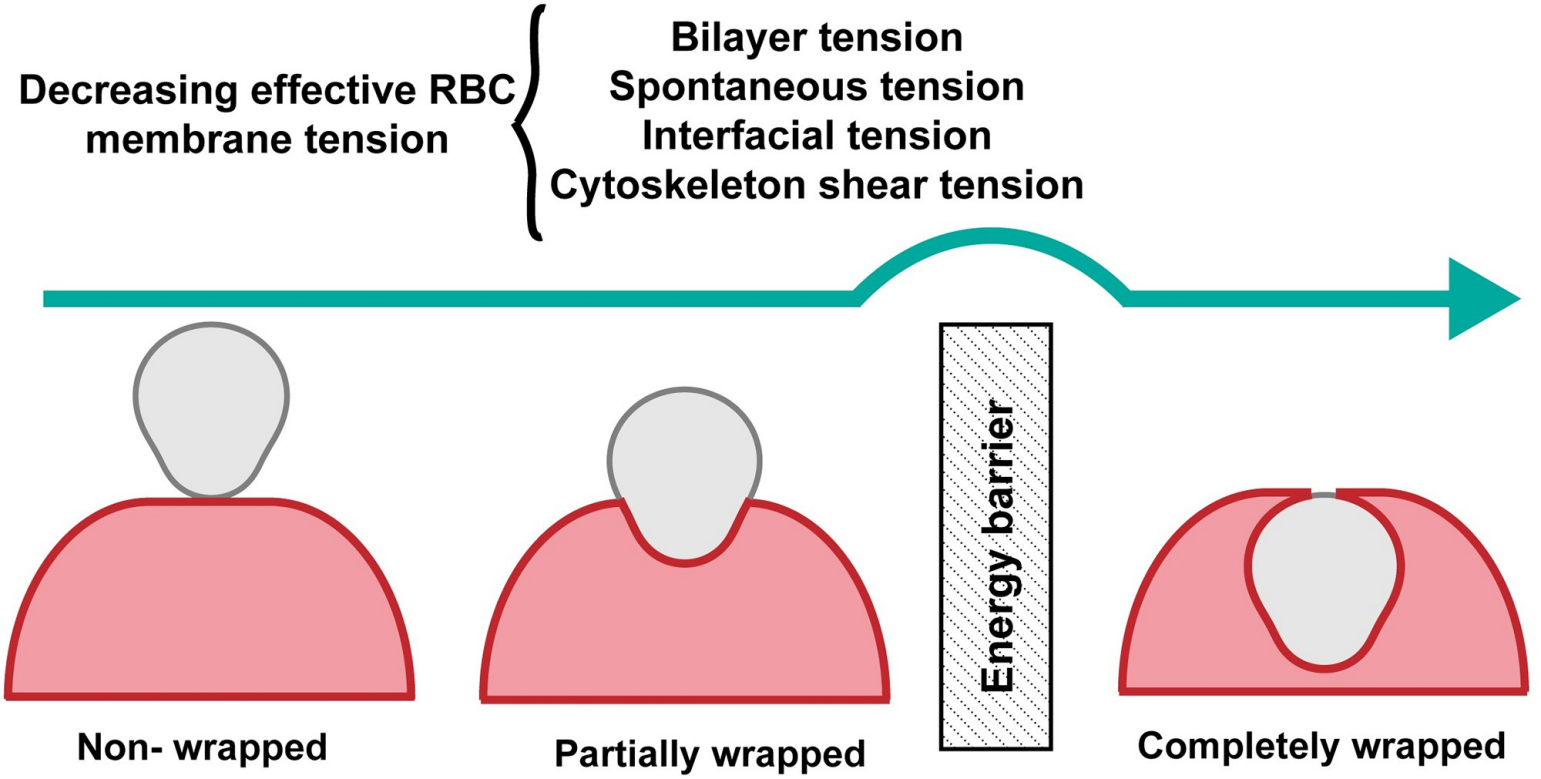
High tension of the RBC membrane acts as a protective mechanism against malaria invasion. Tension of the RBC membrane, including the bilayer tension, induced spontaneous tension, interfacial tension, and the cytoskeleton-induced shear tension generates a wrapping energy barrier and inhibiting malaria invasion.

In this study, we present a mathematical framework for investigating the role of tension of the RBC membrane, consisting of a lipid bilayer and a cytoskeleton layer, in governing the energy landscape of merozoite entry. Here, we modeled the lipid bilayer as an incompressible elastic shell that can bend and the cytoskeleton as an incompressible triangular elastic network (WLC model proposed by Feng el al. [70]) that can undergo shear deformation [46, 70]. Our results show that increasing the effective tension of the RBC membrane generates a wrapping energy barrier, which can hinder the merozoite invasion (Fig 8). The effective tension of the RBC membrane, as summarized in Fig 8 includes the RBC bilayer tension resulting from lipid incompressibility, the cytoskeleton shear tension due to its resistance to deformation, the induced spontaneous tension arising from asymmetry in the lipid distribution, and the induced interfacial tension at the lipid/protein phase separated boundaries (Fig 8).

The presence of an energy barrier in the membrane wrapping process is in agreement with the concept of a membrane tension threshold required for successful malaria invasion proposed in a recent study by Kariuki et al. [22]. The height of the energy barrier depends on the effective tension of the RBC membrane. Particularly, we found that the resting length of the spectrin has a significant contribution in regulating the height of the energy barrier and impeding malaria invasion (Figs 3 and 4). Based on our results, the effective tension threshold needed for inhibiting malaria invasion (*i*) displays an almost linear relationship with the merozoite adhesion to the RBC surface (Fig 3), (*ii*) exhibits a non-monotonic trend with respect to spontaneous tensions, with a slight increase followed by a decrease as spontaneous tension increases (Fig 5), and (*iii*) undergoes a sharp transition from large to small values under high interfacial tensions (Fig 6). These results from our numerical calculations are supported by the analytical expression for membrane wrapping of a spherical particle with no cytoskeleton layer (Eqs S21-S23). By integrating membrane mechanics and signaling dynamics within the RBC cytoskeleton, future studies can further investigate the relationship between the membrane’s biophysical properties and the biochemical processes. For example, the linear relationship between the merozoite adhesion strength and the tension threshold necessary for a complete wrapping can be interpreted as an intrinsic protective response of RBCs due to the activation of the cytoskeleton through receptor-ligand interactions [13, 125].

Taking into account the energy associated with the cytoskeletal deformation in the membrane wrapping process, we found a substantial reduction in the tension threshold for impeding malaria invasion (Figs 3–5). Also, our results show that the shear energy of the cytoskeleton diverges at the full wrapping state (Fig 3). This substantial energy barrier could be overcome by SNARE proteins that interact with tethering factors on the RBC membrane to facilitate the formation of a parasitophorous vacuole [126–128]. For example, disrupting Syntaxin (Stx)-like SNARE protein 12 has been shown to result in a reduction of mature microneme and rhoptry proteins and a significant deficiency in the parasite’s invasion capacity [126]. Several experimental studies have also investigated the role of the RBC cytoskeleton in mediating merozoite invasion [129–131]. In particular, in support of the local disassembly of the cytoskeleton, it has been shown that the success of malaria invasion depends on intracellular ATP hydrolysis and cytoskeleton reorganization [129–131]. We also show the correlation between the shear tension of the cytoskeleton and the three main characteristics of the spectrin network– the natural length (*L*_0_), the maximum length (*L*_max_), and the persistence length of spectrin (*p*)– in membrane wrapping progression (Fig 4). Based on our results, increasing *L*_0_ inhibits the invasion, while larger *L*_max_ and *p* facilitate complete entry (Fig 4). Future models should consider the molecular organization of the actin-spectrin cytoskeleton layer [35, 132, 133].

Another key aspect of malaria invasion is the role of parasite actomyosin motor forces in promoting complete entry [14, 134]. One advantage of our mathematical framework is that it can be used to estimate the minimum actomyosin motor forces required for a complete invasion while taking into account the contribution of membrane wrapping energy during merozoite entry (Fig 8). Based on our calculations, the resistance of the cytoskeleton layer against deformation results in a significant increase (∼ three orders of magnitude) in the actomyosin force needed for complete invasion. (Fig 8). For instance, in a case of a tensionless bilayer, with no adhesion energy, no spontaneous tension, and no interfacial tension, a minimum axial force of *F*_*z*_ ∼ 6 nN is required to deform the RBC cytoskeleton and successfully enter the host cell (Fig 8A). Assuming that each single motor domain of the malaria parasite produces an average force of ∼ 6.5 pN [135], a minimum of ∼ 920 motor domains are required to generate *F*_*z*_ ∼ 6 nN for a successful invasion. This can provide an opportunity for future studies to measure the number of active parasite motors and their relationship with the RBC membrane during malaria invasion.

The study of protection against malaria invasion has been a long-standing area of research [11–14, 16]. Scientists have been exploring various immune responses and genetic factors to better understand and prevent this dangerous disease that kills nearly half a million people every year [136–138]. The role of RBC membrane tension in impeding malaria invasion is a new and highly intriguing concept. We believe our theoretical framework can provide insight into the mechanical aspects of the invasion, providing an opportunity for developing a novel and efficient mechanism to protect against severe malaria infection. For example, the presence of neutralizing antibodies could provide additional resistance to infection, while antibodies can act as obstacles to the membrane wrapping mechanism [139, 140]. In future studies, the role of ligand inhibitors in blocking membrane wrapping can be incorporated as an extra tension contribution to our framework [139]. Furthermore, the red blood cell (RBC) membrane is characterized by a heterogeneous lipid composition, including lipid raft microdomains [141]. This heterogeneity in the lipid composition can induce a distribution of spontaneous tension across the membrane, which based on our results, could serve as a location preference for malaria invasion. Consistently, it has been shown that malarial vacuolar invaginations are enriched with the integral raft protein flotillin-1 [142]. Additionally, we have demonstrated the key contribution of the RBC cytoskeleton in inhibiting merozoite entry, which might explain the malaria protection observed in cases with mutations causing cytoskeleton rearrangements, such as individuals with sickle cell anemia and ovalocytosis [38–40, 125]. We believe these findings can be an important step toward developing efficient antimalarial therapeutic or vaccine-based strategies.

Despite the agreement with experimental data, we acknowledge some limitations and simplifying assumptions of our model. We used a homogeneous contact potential to model the adhesion energy between the merozoite surface and erythrocyte membrane adhesion strength. However, this adhesion energy depends on the number of receptors on the RBC surface, the specific receptor-ligand binding energy, and the configurational entropy of both bound and free receptors [139, 143, 144]. Future efforts will focus on including the dynamics of the receptor-ligand interactions during malaria invasion. Additionally, we modeled the malaria merozoite as a rigid particle adhered to an elastic lipid bilayer. However, for a more general quantitative model, the bending energy of both merozoite and lipid bilayer should be considered [145, 146]. Specifically, we limited our model to axisymmetric shapes, while previous studies have shown that the wrapping of soft nanoparticles is associated with a morphological change and a dramatic symmetry breaking [146]. This requires the development of new frameworks for investigating wrapping and the mechanics of receptor-mediated endocytosis of asymmetric-shaped merozoites [96].

## Supporting information

S1 Text Supplementary material text containing Figs A-D. (PDF)
