# Supplementary material for: Effective cell membrane tension protects red blood cells against malaria invasion
Source: PLoS Comput Biol. 2023 Dec 4;19(12):e1011694. doi: 10.1371/journal.pcbi.1011694 (PMC10721198; doi:10.1371/journal.pcbi.1011694)
Supplement: S1 Text — (PDF) [file pcbi.1011694.s001.pdf]

Supplementary material for "Effective cell membrane tension protects red blood cells against malaria invasion"

H. Alimohamadi[1§] and P. Rangamani[1*]

[1]Department of Mechanical and Aerospace Engineering, University of California San Diego, CA 92093, USA
[§]Current affiliation: Department of Bioengineering, University of California Los Angeles, Los Angeles, CA 90025
[*]To whom correspondence should be addressed. E-mail: prangamani@ucsd.edu

Specialization to axisymmetric coordinates

For computational ease, we specialize the membrane and merozoite to axisymmetric coordinates. We parameterize a surface of revolution as $\mathbf{r}(s) = r(s)\mathbf{e}_r + z(s)\mathbf{k}$, where s is the arclength along the curve, $r(s)$ is the radial distance from the axis of rotation, and $z(s)$ is the elevation from the reference plane. In axisymmetric coordinates, the integral over the adhered area and the integral over interfacial length in Eq. 7 simplify as

$$\int_{A_{ad}} da = 2\pi \int_0^{s_{max}} r ds \quad \text{and} \quad \gamma \oint_{\partial l} dl = 2\pi\gamma r. \tag{S1}$$

where s_{max} is the maximum membrane arclength that adheres to the merozoite. Additionally, for axisymmetric coordinates, the principal extension ratios can then be written as

$$\lambda_1 = \frac{ds}{ds_0} = \frac{1}{s_0'} \quad \text{and} \quad \lambda_2 = \frac{r}{s_0}, \tag{S2}$$

where $(.)' = \frac{d(.)}{ds}$ and s_0 is the arclength along the undeformed shape of the axisymmetric skeleton mapping to an unknown position s on the deformed shape.

The egg shape of an archetypal merozoite in an axisymmetric coordinate can be parametrized as [1]

$$(X^2 + Y^2 + Z^2) = R_a X^3 + (R_a - R_b)X(Y^2 + Z^2), \text{ with } R_a = 1\mu\text{m}, R_b = 0.7\mu\text{m}, \tag{S3}$$

where

$$\begin{aligned} X(\phi,\theta) &= \Big(\frac{(2R_a - R_b)\sin(\theta)}{4} - \frac{R_b\sin(2\theta)}{8}\Big)\cos(\phi), \\ Y(\phi,\theta) &= \Big(\frac{(2R_a - R_b)\sin(\theta)}{4} - \frac{R_b\sin(2\theta)}{8}\Big)\sin(\phi), \\ Z(\phi,\theta) &= -\Big(\frac{2R_a - R_b\cos(\theta)}{4}\Big)\cos(\theta), \end{aligned} \tag{S4}$$

where $0 < \phi < 2\pi$ and $0 < \theta < \pi$. Using Eq. S4, the radius of merozoite (R) as a function of angle θ can be written as

$$R(\theta) = \sqrt{X^2+Y^2+Z^2} = \sqrt{\Big(\frac{(2R_a - R_b)\sin(\theta)}{4} - \frac{R_b\sin(2\theta)}{8}\Big)^2 + \Big(\frac{2R_a + R_b\cos(\theta)}{4}\Big)^2\cos^2(\theta)}. \quad \text{(S5)}$$

Having the radius of merozoite, we can find the radial distance from the axis of rotation (r) and the elevation from the reference plane (z) given by

$$\begin{aligned} r(\theta) &= R(\theta)\sin(\theta) \\ z(\theta) &= -R(\theta)\cos(\theta). \end{aligned} \quad \text{(S6)}$$

Using Eq. S6 and the definition of axisymmetric coordinates, we have

$$\begin{aligned} k_1 &= -\frac{dz}{d\theta}\frac{1}{r\sqrt{(dr/d\theta)^2+(dz/d\theta)^2}} \\ k_2 &= \frac{dz/d\theta \times d^2r/d\theta^2 - d^2z/d\theta^2 \times dr/d\theta}{[(dr/d\theta)^2+(dz/d\theta)^2]^{3/2}}, \end{aligned} \quad \text{(S7)}$$

where k_1 and k_2 are the surface principal curvatures and $\frac{ds}{d\theta} = \sqrt{(dr/d\theta)^2+(dz/d\theta)^2}$. Eq. S7 allows us to find the mean curvature H along the merozoite surface as a function of θ given as

$$2H(\theta) = k_1 + k_2, \quad \text{(S8)}$$

where $\dot{(.)} = \frac{d(.)}{d\theta}$. The integral over the adhered area (Eq. S1) and the extension ratios (Eq. S2) can also be calculated as a function of θ

$$\int_{A_{ad}} da = 2\pi\int_0^{\theta_{max}} r\sqrt{(dr/d\theta)^2+(dz/d\theta)^2}d\theta, \quad \text{(S9a)}$$

$$\lambda_1 = \frac{\sqrt{(dr/d\theta)^2+(dz/d\theta)^2}d\theta}{ds_0} = \frac{\sqrt{(dr/d\theta)^2+(dz/d\theta)^2}}{\dot{s_0}} \quad \text{and} \quad \lambda_2 = \frac{r(\theta)}{s_0(\theta)}, \quad \text{(S9b)}$$

where θ_{max} is the maximum wrapping angle. Assuming that actomyosin motors apply forces tangentially along the membrane surface, in axisymmetric coordinates, the net radial force ($\mathbf{f}_r = f\cos(\theta)$) is zero. Thus, only the axial component of actomyosin forces ($\mathbf{f}_z = f\sin(\theta)$) pushes the merozoite forward and the work on the membrane (Eq. 3) is simplified as

$$E_f = \int_0^{\theta_{max}} \underbrace{\Big(2\pi r f\sin(\theta)\sqrt{(dr/d\theta)^2+(dz/d\theta)^2}\Big)}_{\text{Axial force in z direction (Fz)}} \underbrace{\Big(R\big(1-\cos(\theta)\big)\Big)}_{\text{Axial displacement in z direction}} d\theta. \quad \text{(S10)}$$

Using Eqs. S1, S9a, and S10, the change in the energy of bilayer/cytoskeleton due to the adhesion of merozoite and deformation bilayer/cytoskeleton (Eq. 7) can be written as a function θ

$$\begin{aligned} \Delta E(\theta) = 2\pi\int_0^{\theta_{max}} \Big[r\Big(2\kappa(H(\theta)-H_0)^2 + \sigma - \omega - f\sin(\theta)R(\theta)\big(1-\cos(\theta)\big) + W_c(\theta)\Big)\sqrt{R(\theta)^2+\dot{R}(\theta)^2}\Big]d\theta \\ +2\pi\gamma r(\theta_{max}) - \pi\sigma_{\text{bilayer}}\Big(\int_0^{\theta_{max}} 2r\sqrt{(dr/d\theta)^2+(dz/d\theta)^2}d\theta - r(\theta_{max})^2\Big), \end{aligned} \quad \text{(S11)}$$

where

$$W_c = \underbrace{\frac{2c_\beta}{3x_0^2} \sum_{\substack{\phi=n\pi/6 \\ n\in\{1,2,..6\}}} x_0^2\big(\lambda_1^2\cos(\phi)^2 + \lambda_2^2\sin(\phi)^2\big)\frac{3-2x_0\sqrt{\lambda_1^2\cos(\phi)^2+\lambda_2^2\sin(\phi)^2}}{1-x_0\sqrt{\lambda_1^2\cos(\phi)^2+\lambda_2^2\sin(\phi)^2}}}_{\text{Entropic energy of spectrin filaments orientations}} + \underbrace{c_\beta\frac{4x_0^2-9x_0+6}{(1-x_0)^2}}_{\text{Steric interactions}}. \tag{S12}$$

Here, in Eq. S11, we assumed that the force density applied by the actomyosin motor (f) is constant all along the area of adhered merozoite.

Incompressible bilayer and cytoskeleton

Let us assume that a flat circular patch of a lipid bilayer and relaxed cytoskeleton with radius (s_0) deformed to fit the merozoite contour in the adhesive region. Thus, for an incompressible bilayer/cytoskeleton, the area conservation can be written as

$$\pi s_0^2 = 2\pi\int_0^{\theta_{max}} r\sqrt{(dr/d\theta)^2+(dz/d\theta)^2}d\theta. \tag{S13}$$

Eq. S16 allows us to find s_0 and calculate the extension ratios using Eq. S9b, which simplifies as

$$\lambda_1 = \frac{\sqrt{(dr/d\theta)^2+(dz/d\theta)^2}}{\dot{s_0}} = \frac{s_0(\theta)}{r(\theta)} \quad \text{and} \quad \lambda_2 = \frac{r(\theta)}{s_0(\theta)} = \frac{1}{\lambda_1}, \tag{S14}$$

which is consistent with zero local area strain ($\alpha = \lambda_1\lambda_2 - 1 = 0$) for an incompressible bilayer/cytoskeleton [2]. Additionally, for an incompressible cytoskeleton, the shear modulus μ is simplified as [3]

$$\mu = \frac{4c_\beta}{3x_0(\lambda_1^2-\lambda_2^2)}\Big(c_0 + c_1(\lambda_1-1) + \frac{\lambda_1}{4(1-\lambda_1 x_0)^2}\Big), \tag{S15}$$

where $c_0 = \frac{-1}{4(1-x_0)^2}$ and $c_1 = \frac{48x_0^4-153x_0^3+171x_0^2-71x_0+1}{4(x_0-1)^3}$.

Numerical implementation

For an egg shape merozoite parametrized by Eq. S3, we numerically calculate the change in the energy of the bilayer/cytoskeleton as a function of wrapping angle θ (Eq. S11). Then, for any given set of constant parameters, we find an angle θ^* at which the invasion state becomes an energy minimum.

Incompressible bilayer and cytoskeleton

Let us assume that a flat circular patch of a lipid bilayer and relaxed cytoskeleton with radius (s_0) deformed to fit the merozoite contour in the adhesive region. Thus, for an incompressible bilayer/cytoskeleton, the area conservation can be written as

$$\pi s_0^2 = 2\pi \int_0^{\theta_{max}} r\sqrt{(dr/d\theta)^2 + (dz/d\theta)^2} d\theta. \tag{S16}$$

Eq. S16 allows us to find s_0 and calculate the extension ratios using Eq. S9b, which simplifies as

$$\lambda_1 = \frac{\sqrt{(dr/d\theta)^2 + (dz/d\theta)^2}}{\dot{s_0}} = \frac{s_0(\theta)}{r(\theta)} \quad \text{and} \quad \lambda_2 = \frac{r(\theta)}{s_0(\theta)} = \frac{1}{\lambda_1}, \tag{S17}$$

which is consistent with zero local area strain ($\alpha = \lambda_1\lambda_2 - 1 = 0$) for an incompressible bilayer/cytoskeleton Additionally, for an incompressible cytoskeleton, the shear modulus μ is simplified as

$$\mu = \frac{4c_\beta}{3x_0(\lambda_1^2 - \lambda_2^2)}\Big(c_0 + c_1(\lambda_1 - 1) + \frac{\lambda_1}{4(1-\lambda_1 x_0)^2}\Big), \tag{S18}$$

where $c_0 = \frac{-1}{4(1-x_0)^2}$ and $c_1 = \frac{48x_0^4 - 153x_0^3 + 171x_0^2 - 71x_0 + 1}{4(x_0-1)^3}$.

Numerical implementation

For an egg shape merozoite parametrized by Eq. S3, we numerically calculate the change in the energy of the bilayer/cytoskeleton as a function of wrapping angle θ (Eq. S11). Then, for any given set of constant parameters, we find an angle θ^* at which the invasion state becomes an energy minimum.

Analytical approximations

In this section, we explore the analytical solution for the minimum energy state, ignoring the effects of membrane cytoskeleton energy and modeling the merozoite as a spherical particle with radius a. In this condition, the change in the energy of the system (Eq. S11) can be written as

$$\begin{aligned}\Delta E(\theta) = 4\pi\kappa a^2(1/a - H_0)^2 y + \pi\sigma a^2 y^2 - 2\pi\omega a^2 y + 2\pi\gamma\sqrt{y(2-y)} - 2\pi\kappa H_0^2 a^2(2y - y^2)\\ -\frac{\pi f a^3}{3}\big(3\cos^{-1}(1-y) + \sqrt{y(2-y)}(2y^2 - y - 3)\big),\end{aligned} \tag{S19}$$

where $y = 1 - \cos(\theta)$. By taking $\frac{\partial \Delta E}{\partial y} = 0$, we have

$$\begin{aligned}2\kappa(1 - 2H_0 a) - \omega a^2 + (\sigma a^2 + 2\kappa H_0^2 a^2)y + \gamma a\frac{1-y}{\sqrt{y(2-y)}}\\ -f a^3 y\sqrt{y(2-y)} = 0.\end{aligned} \tag{S20}$$

Considering our definition for a completely wrapped state ($\theta^* > \pi/2$), we can find the transition condition to the completely wrapped state by setting $y = 1$ in Eq. S20. Below, we simplified Eq. S20 for different conditions.

- **Case 1: Relationship between lipid bilayer tension and adhesion strength**
  Considering the condition that $\gamma = 0$, $f = 0$, and $H_0 = 0$, Eq. S20 simplifies as

$$\sigma = \omega - 2\kappa/a^2. \tag{S21}$$

Eq. S21 suggests that the particle can get fully wrapped with increasing adhesion strength.

- **Case 2: Relationship between lipid bilayer tension and spontaneous curvature**
  Considering the condition that $\gamma = 0$ and $f = 0$, Eq. S20 gives

$$\sigma = \omega - 2\kappa(1/a - H_0)^2. \tag{S22}$$

  Based on Eq. S22, when the induced spontaneous curvature is smaller than the curvature of the particle ($H_0 < 1/a$), the induced spontaneous curvature assists the progress of complete particle wrapping. However, larger spontaneous curvatures ($H_0 > 1/a$) impede the complete wrapping transition.

- **Case 3: Relationship between lipid bilayer tension and interfacial forces**
  As can be seen, Eq. S20 has a symmetric barrier at $\theta = \pi/2$ (the line tension term vanishes for $y = 1$). Thus, to find the analytical approximation, we set $y = 1 \pm \epsilon$, where ϵ is a small number, and expanded the Eq. S20 until the first order for the case that $f = 0$ and $H_0 = 0$

$$\theta < \pi/2 \rightarrow y = 1 - \epsilon \rightarrow \sigma = (\omega - 2\kappa/a^2)(1 + \epsilon) - \gamma\epsilon/a, \tag{S23a}$$
$$\theta > \pi/2 \rightarrow y = 1 + \epsilon \rightarrow \sigma = (\omega - 2\kappa/a^2)(1 - \epsilon) + \gamma\epsilon/a. \tag{S23b}$$

  Based on Eqs. S23, in the first half of wrapping ($\theta < \pi/2$), a line tension prevents the complete membrane wrapping process. However, once the equator is passed ($\theta > \pi/2$), a line tension accommodates the particle encapsulation. It should be mentioned that with no line tension ($\gamma = 0$), a non-wrapped state ($\theta^* = 0$) is always a local minimum of $\Delta E(\theta)$. However, the line tension and actomyosin force energy terms scale as $\sqrt{y}$ and their derivatives diverge at $\theta = 0$. This means that a line tension can create an energy barrier with no minimum energy state between $0 \leqslant \theta \leqslant \pi$ in which the particle even does not adhere to the membrane.

- **Case 4: Motor forces required for a complete wrapping as a function of membrane physical properties**
  To calculate the minimum motor forces that are required for a complete particle wrapping (based on our definition $\theta^* > \pi/2$), we substitute $y = 1 + \epsilon$ in Eq. S20 and find the force density (f) as

$$fa^3 = 2\kappa(1 - 2H_0 a)(1 - \epsilon) - \omega a^2(1 - \epsilon) + (\sigma a^2 + 2\kappa H_0^2 a^2) - \gamma a\epsilon. \tag{S24}$$

  The total force in the z direction (F_z) is obtained as

$$F_z = 2\pi \int_0^{\theta_{max}} rf \sin(\theta) d\theta = \pi a^2 f\big(\cos^{-1}(1 - y) - \sqrt{y(2 - y)}(1 - y)\big). \tag{S25}$$

  Substituting Eq. S24 into Eq. S25 for a complete wrapping condition ($y = 1 + \epsilon$), we have

$$F_z = \pi\Big(2\kappa a(1/a - H_0)^2 - \omega a + \sigma a - \big(2\kappa(1/a - H_0) - \omega a + \gamma\big)\epsilon\Big)(\pi/2 + 2\epsilon). \tag{S26}$$

  Based on Eq. S26, for a tensionless membrane ($\sigma = 0$), with no adhesion energy ($\omega = 0$), no line tension ($\gamma = 0$), and no spontaneous curvature ($H_0 = 0$), a minimum force of F_z = 3 pN is required for a complete wrapping of a spherical particle. This is consistent with the calculated magnitude of actomyosin forces required for a merozoite invasion by Dasgupta et al [1]. It should be mentioned that Eq. S26 is derived for the minimum axial force needed for a complete invasion. This means if the right hand side of Eq. S26 becomes negative, the physical forces are enough to push the merozoite into the RBC and thus F_z = 0.

Supplementary Figures

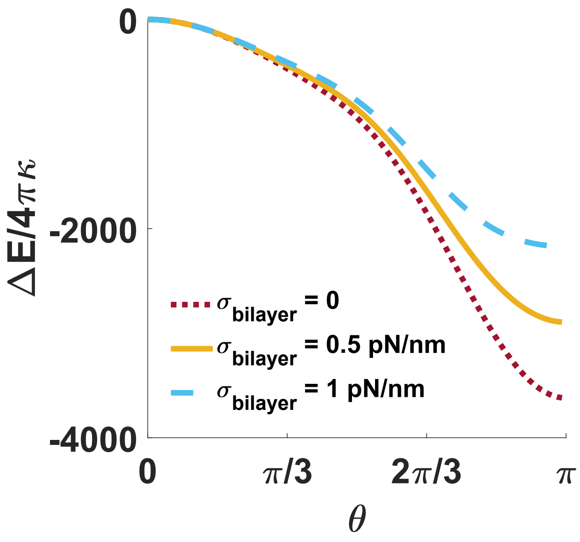

Figure A: The change in the energy of the RBC bilayer with no cytoskeleton layer as a function of wrapping angle (θ) for a fixed ω = 2.5 pN/nm and three different bilayer tension. The change in the energy is minimized in a completely wrapped state ($\theta^* = \pi$) independent of the magnitude of the bilayer tension.

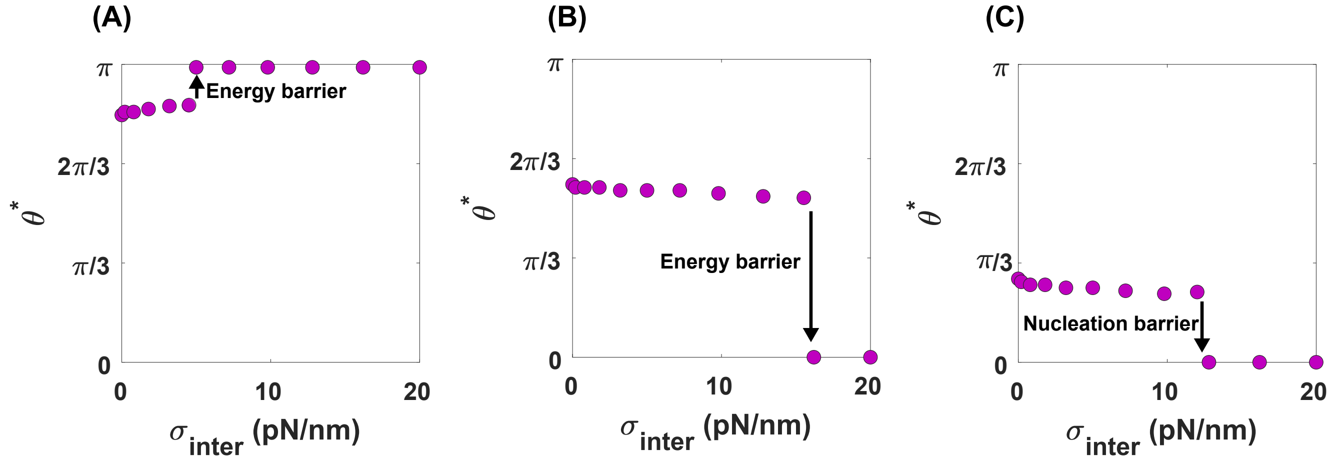

Figure B: θ^* as a function of interfacial tension for wrapping of an egg-shaped merozoite without the cytoskeleton layer. **(A)** A discontinuous transition from $\theta^* \sim 5\pi/6$ to a full wrapped state ($\theta^* = \pi$) with an increase in the magnitude of interfacial tension, ω = 1 pN/nm and σ_{bilayer} = 0.6 pN/nm. **(B)** A discontinuous transition from $\theta^* \sim 5\pi/9$ to a non-adhered state with an increase in the magnitude of interfacial tension, ω = 0.4 pN/nm and σ_{bilayer} = 0.6 pN/nm. **(C)** A discontinuous transition from a partially wrapped state to a non-adhered state with an increase in the magnitude of interfacial tension, ω = 0.5 pN/nm and σ_{bilayer} = 1 pN/nm.

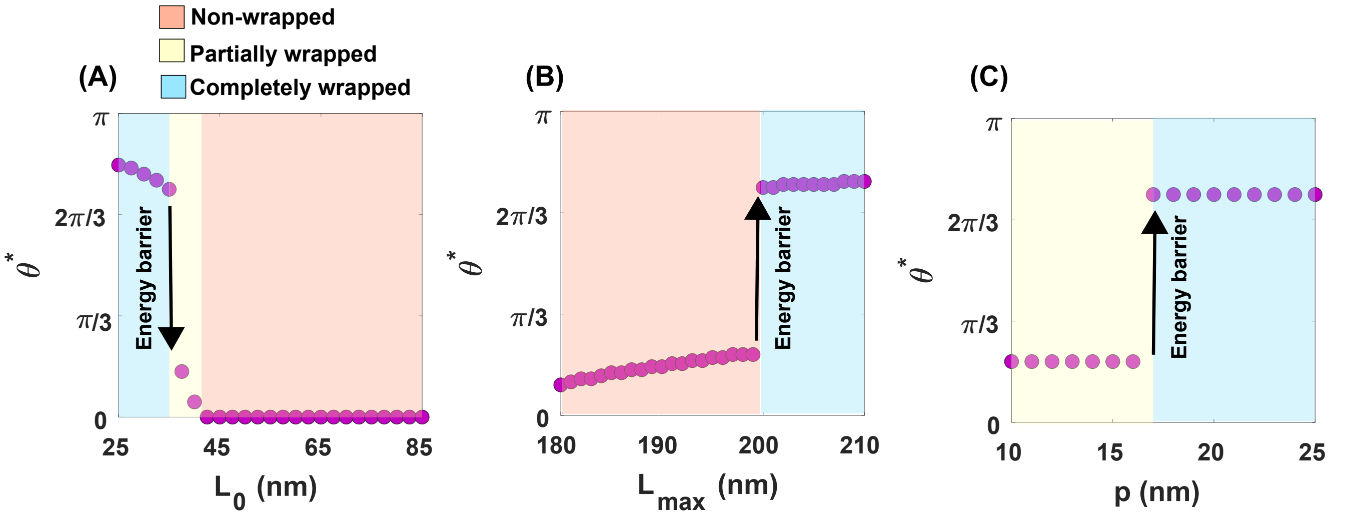

Figure C: The effects of physical properties of the cytoskeleton on the efficiency of malaria invasion, $\sigma_{bilayer}$ = 0.63 pN/nm. **(A)** A discontinuous transition from a completely to a partially wrapped state followed by a continuous transition from a partially to a non-wrapped wrapped state with increasing L_0 from 25 nm to 85 nm. σ = 0.1 pN/nm, ω = 0.8 pN/nm, p = 25 nm, and L_{max} = 200 nm. **(B)** A continuous transition from a partially to a completely wrapped state followed by a discontinuous transition from a partially to a completely wrapped state with increasing L_{max} from 180 nm to 210 nm. σ = 0.1 pN/nm, ω = 0.8 pN/nm, p = 25 nm, and L_0 = 35 nm. **(C)** A discontinuous transition from a partially wrapped to a completely wrapped state with increasing the persistence length of spectrin p. σ = 0.1 pN/nm, ω = 0.8 pN/nm, L_0 = 35 nm, and L_{max} = 200 nm.

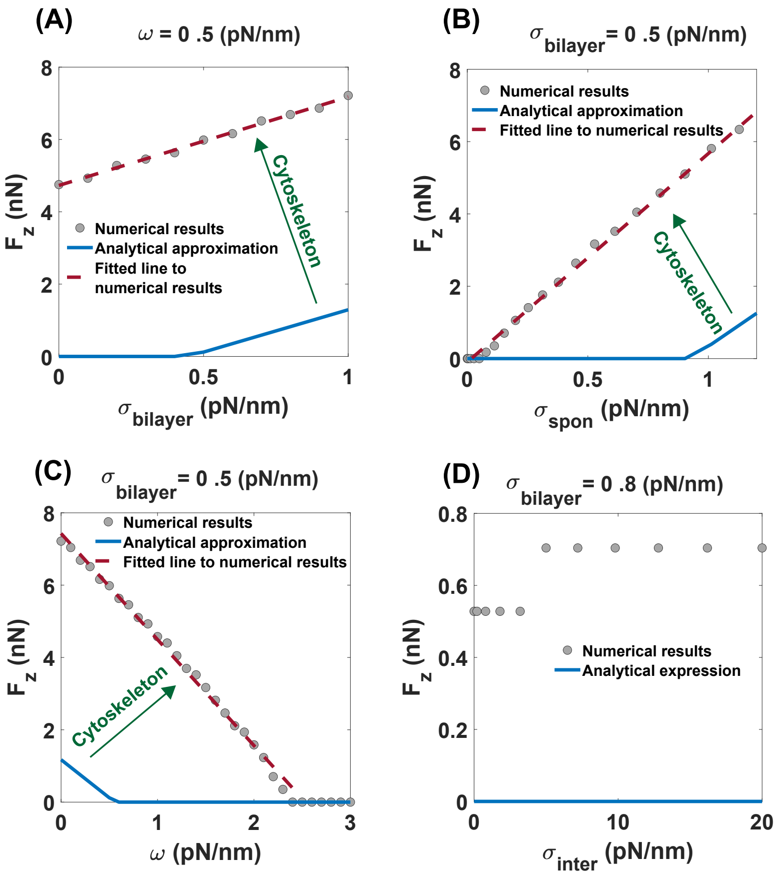

Figure D: Minimum axial force (F_z) required for a complete merozoite entry as a function of **(A)** bilayer tension, **(B)** spontaneous tension, **(C)** adhesion strength, and **(D)** interfacial tension. p = 25 nm, L_0 = 35 nm, and L_{max} = 200 nm. The gray circles show the results that we obtained from the energy minimization (Eq. S11). The dotted line represents the fitted curves and the solid blue line indicates the analytical approximation for the motor-driven force (Eq. S26). The green arrow demonstrates the increase in the magnitude of the axial force compared to the analytical approximations because of the cytoskeleton resistance against deformation. **(A)** F_z increases as a linear function of bilayer tension. The dashed line shows the linear dependence on the bilayer tension by fitting to a line (Aσ_{bilayer}+B), where A = 2.48 and B = 4.73 with R^2 = 0.99. **(B)** F_z varies as a linear function of spontaneous tension. The dashed line shows a linear dependence on the spontaneous tension by fitting to a line (Aσ_{spon}+B), where A = 5.7, B =-0.09 with R^2 = 0.99. **(C)** F_z decreases as a linear function of adhesion strength. The dashed line shows the linear dependence on the adhesion strength by fitting to the line (Aω+B), where A = -2.93 and B = 7.4 with R^2 = 0.99. **(D)** Switch-like increases in axial force from F_z = 0.52 nN to F_z = 0.7 nN with increasing the magnitude of interfacial tension.

References

[1] S. Dasgupta, T. Auth, N. S. Gov, T. J. Satchwell, E. Hanssen, E. S. Zuccala, D. T. Riglar, A. M. Toye, T. Betz, J. Baum, *et al.*, "Membrane-wrapping contributions to malaria parasite invasion of the human erythrocyte," *Biophysical journal*, vol. 107, no. 1, pp. 43–54, 2014.

[2] E. A. Evans, *Mechanics and thermodynamics of biomembranes*. CRC press, 2018.

[3] Z. Feng, R. E. Waugh, and Z. Peng, "Constitutive model of erythrocyte membranes with distributions of spectrin orientations and lengths," *Biophysical Journal*, vol. 119, no. 11, pp. 2190–2204, 2020.
